# On the Reconstruction of Text Phylogeny Trees: Evaluation and Analysis of Textual Relationships

**DOI:** 10.1371/journal.pone.0167822

**Published:** 2016-12-19

**Authors:** Guilherme D. Marmerola, Marina A. Oikawa, Zanoni Dias, Siome Goldenstein, Anderson Rocha

**Affiliations:** University of Campinas (Unicamp), Institute of Computing, Campinas, São Paulo, Brazil; Medical University of South Carolina, UNITED STATES

## Abstract

Over the history of mankind, textual records change. Sometimes due to mistakes during transcription, sometimes on purpose, as a way to rewrite facts and reinterpret history. There are several classical cases, such as the logarithmic tables, and the transmission of antique and medieval scholarship. Today, text documents are largely edited and redistributed on the Web. Articles on news portals and collaborative platforms (such as *Wikipedia*), source code, posts on social networks, and even scientific publications or literary works are some examples in which textual content can be subject to changes in an evolutionary process. In this scenario, given a set of near-duplicate documents, it is worthwhile to find which one is the original and the history of changes that created the whole set. Such functionality would have immediate applications on news tracking services, detection of plagiarism, textual criticism, and copyright enforcement, for instance. However, this is not an easy task, as textual features pointing to the documents’ evolutionary direction may not be evident and are often dataset dependent. Moreover, side information, such as time stamps, are neither always available nor reliable. In this paper, we propose a framework for reliably reconstructing text phylogeny trees, and seamlessly exploring new approaches on a wide range of scenarios of text reusage. We employ and evaluate distinct combinations of dissimilarity measures and reconstruction strategies within the proposed framework, and evaluate each approach with extensive experiments, including a set of artificial near-duplicate documents with known phylogeny, and from documents collected from Wikipedia, whose modifications were made by Internet users. We also present results from qualitative experiments in two different applications: text plagiarism and reconstruction of evolutionary trees for manuscripts (stemmatology).

## Introduction

Content redistribution over the Web, by lawful or unlawful means, has recently attracted the attention in several research fields, such as digital forensics, copyright enforcement, security, and social network monitoring. Once one image or video is posted by a user in a social network, or a text is posted in a blog, it is prone to be downloaded and modified by several other users in an uncontrolled manner. In addition, each new version can be downloaded and modified again, creating several variants of the original object, in different ramifications. Finding the dependencies among these variants, that is, reconstructing the *evolutionary tree* associated with them can help us to understand how some information—such as news—spreads through the Web, or identify criminals by tracing abusive documents to their source.

*Multimedia phylogeny* is a research field that studies the problem of discovering phylogenetic dependencies in digital media. It was first defined by Dias et al. [1], and currently includes several approaches dealing mainly with images and videos [2–8]. In this field, the main goal is to find the transformations and the parameters that generated a given set of near-duplicate digital objects. Following the same terminology used in multimedia phylogeny for other media types, in this work a near-duplicate document (or a copy) is defined as a transformed version of one original document (*root*) that remains recognizable [9].

One can visualize the procedure of reconstructing a media phylogeny tree with a direct analogy to the evolutionary process in Biology: similarly to how living organisms evolve, digital objects can be changed to different versions of themselves over time, creating several new branchings. However, instead of using a Steiner tree, solutions presented in multimedia phylogeny use a minimum spanning tree for reconstructing the phylogeny, since it is considered only the objects present in the set being analyzed, and it is not possible to include unknown internal nodes to reduce the length of the tree.

**Fig 1:**
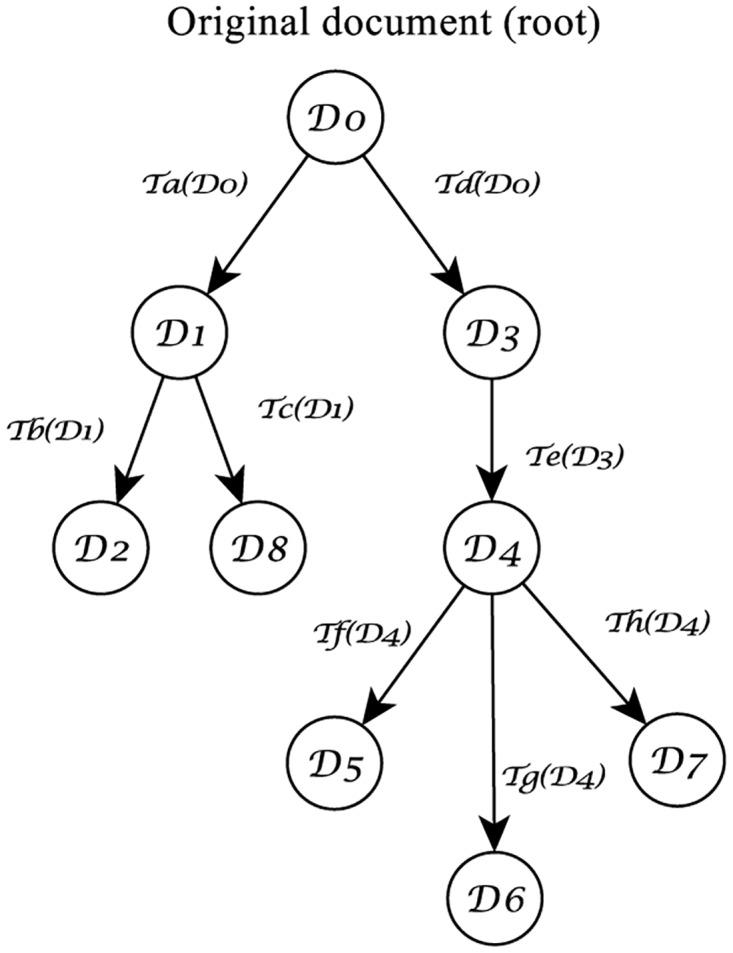
An example of a near-duplicate phylogeny tree. $D_{0}$ is the original document (or root of the tree), from which several other versions are created following a set $T_{\alpha }$ of transformations.

In this paper, we focus on approaches for *text phylogeny*, to re-create the history and evolutionary process of a given set of near-duplicate text documents following a similar approach developed in multimedia phylogeny, in which we consider only the cases in which all objects belonging to the tree are known. Fig 1 illustrates the case of an original document $D_{0}$, and the tree with its near duplicates, where each descendant document is created after applying a set $T_{\alpha }\left(D_{i}\right)$ of transformations. For text documents, these transformations may include synonym exchange, and insertion or removal of sentences, misspelings or modifiers (adjectives and adverbs), for instance. Although all documents in this tree are copies of $D_{0}$, they are not necessarily copies of each other, since every $D_{i}$ can generate new copies in different branchings.

The attempt to reconstruct phylogenetic relationships within a set of related text documents is a challenge of interest not only on our current digital era. For instance, historians have been studying this problem in handwritten medieval manuscripts [10–12], Babbage was very concerned about errors in logarithmic tables [13], and The Book of Soyga, which surrounds an interesting part of John Dee’s mystical interests [14], had multiple slightly different versions.

Textual criticism and stemmatology have been playing an important role on the reconstruction of original manuscript texts from a set of different copies derived from it. In general, these copies (or surviving variants) appear as a result of loss of data when a manuscript was partially destroyed, or from human errors introduced during the copying process. After analyzing their relationship, these copies are combined in a tree structure called stemma, representing the history of the text, with the original version as the root of the tree [15]. Although the general idea behind stemmatology is similar to our goal in text phylogeny, the challenges faced for each field are slightly different. In stemmatology, there is a concern regarding dealing with incomplete data (missing parts and imperfect copied files) for reconstructing the original text, while in multimedia phylogeny up to this point it is assumed all objects composing the tree are known. Furthermore, more emphasis is given on the inferred structure (the number of copies between the variants), rather than grouping them based on their similarity to infer their relationship [16]. A broader discussion regarding stemmatology is also provided by van Reenen et al. [17].

In text phylogeny, unlike stemmatology, the fundamental aim is to find the relationships among near-duplicate text documents through the analysis of their transformations over time. In this paper, our utmost goal is the reconstruction of an evolutionary or phylogeny tree that represents the lineage and the content evolution of a set of *n* near-duplicate text documents. We assume that all documents in the set being analyzed inherit content from one single ancestor, but each of them may spur any number of copies. Therefore, since we are not targeting the reconstruction of the original text, the absence of part of the document is handled the same way as other textual operations such as the exchange of a word by a synonym, or the appearance of a spelling mistake. In addition, there is no assumption that the original documents’ source is present in the set; instead, we aim at finding the document that most probably is the common ancestor of all objects within the set being analyzed. Therefore, one of the documents in the set is always chosen as the root of the current reconstructed tree, regardless the presence or absence of the original document. Nevertheless, we show in the Experiments and Results section the behavior of our approach for such cases.

Compared to the approaches for image and video phylogeny, the tree reconstruction for text documents is the most challenging part, as devising an asymmetric dissimilarity measure between two text documents, and hence, determining the direction of the modifications, is not an easy task. If such a tree could be correctly reconstructed, it would be helpful in several applications, such as analysis of the evolution of news over time (news tracking) and its influence on readers’ opinion formation process, detection and clustering of plagiarism cases, and finding intertextual connections between classic writers [18], for instance.

### Related work

In the literature, there are several approaches using concepts from evolutionary Biology to study the underlying relationship of non-biological objects. Some examples include the use of phylogeny trees in archeology to reconstruct artifacts’ lineages [19, 20], in name phylogeny to verify if a given name in the set was generated from scratch or derived from another name, and find its variants [21], and in multimedia phylogeny, to represent the structure of transformations and the evolution of near-duplicate images [1, 3–5], videos [2, 6, 8], and audio files [22].

In the scope of textual document analysis, phylogeny trees have been used to analyze the evolution of malicious code, such as computer viruses and other malware [23–25]. Such trees are helpful to assist the development of methods for detecting and classifying upcoming attacks, as well as the investigation of new types of malware.

In Cuadros et al. [26]’s approach, phylogeny trees were used for building visual maps of documents based on their content similarity. In this case, ancestry relationships are built according to the documents content correlation, with the final tree comprising documents sharing the same topic, but not necessarily a common source.

On another category, some works in stemmatic analysis compare the evolution of manuscripts to the evolution and mutations in DNA sequences, and the relationship among a manuscript and its extant versions is also represented by means of a phylogeny tree [10, 17, 27]. In a similar trend, Spencer et al. [12] and Roos and Heikkila [15] evaluate different methods for reconstructing the phylogenetics of manuscript copies created artificially, and whose true phylogeny is known. However, these approaches reconstruct unrooted trees (or are manually rooted), and focus is given to the identification of groups of copies that are closer to each other. Further advances are still necessary to locate these groups within the stemma, to reliably reconstruct large textual traditions, and also detect polytomies (cases when one node has more than two direct descendants).

As the similarity values calculated among text documents cannot be used alone to determine the direction of the modifications, there are only a few approaches which attempt to infer the directionality of the transformations by using some additional information or features that are specific for the target group of documents. For instance, in Ryu et al.’s work [28, 29], the evolutionary tree of plagiarized documents is reconstructed by combining two similarity values: spatial and temporal. While the spatial similarity calculates the content similarity among the documents using a probabilistic model, the temporal similarity uses timestamps to determine the plagiarism direction. However, when dealing with real-world setups, this approach may not be feasible, as the time information is not always available, and its authenticity can be dubious.

Grozea and Popescu [30] proposed a method for automatic detection of the direction of plagiarism using a dotplot-like analysis of character-based n-grams shared by pairs of text documents (encoplot). Although presenting good results, the asymmetry found in the encoplot graphic is measured under the assumption that the probability of having multiple instances of the same n-gram in the remaining text of the source document is higher than in the remaining text of the destination document. In addition, this approach fails when the documents include copied passages that are too short or too close to the beginning of one of the texts, crowded plots, and too short texts. It would be also difficult to apply this method in the context of text phylogeny or in stemmatology, as in both cases the text documents as a whole (and not some excerpts) are very similar to each other, making it difficult to create the clouds of dots.

In source code plagiarism detection, an asymmetric score has been proposed by Ji et al. [31, 32], whose calculation is based on the frequency of keywords in a group of functional-equivalent programs (e.g., programs submitted in an assignment task). An adaptive score matrix is calculated using an asymmetric alignment score, controlled by four parameters: −*α* (matched keywords), *β* (mismatched keywords), *γ* (gap insertion), and *δ* (gap deletion). These parameters work as weights for each of the aforementioned conditions (matching, mismatching, insertion, and deletion of keywords), being dependent on the characteristics of each program group. For instance, it is considered that is more difficult to delete than to insert a keyword to maintain the same function (hence, *γ* < *δ*), and keywords with lower frequency have higher matching score, as they might be more crucial to the functional aspects of the program. Thus, given two programs *P*_*a*_ and *P*_*b*_, *EvolDist*(*P*_*a*_, *P*_*b*_) ≠ *EvolDist*(*P*_*b*_, *P*_*a*_), and if *EvolDist*(*P*_*a*_, *P*_*b*_) < *EvolDist*(*P*_*b*_, *P*_*a*_), it is assumed that *P*_*b*_ is derived from *P*_*a*_.

In solving the directionality problem with asymmetric measures, the aforementioned approaches work with some restrictions or under assumptions that are not suitable to all types of text documents. For instance, source code has special structures that cannot be found in other types of text documents, such as special symbols or reserved words, and unless we have previous knowledge regarding a special feature on the target dataset, it is harder to directly apply these methods in a general way.

## Materials and Methods

In this paper, the term *phylogeny tree* is used to keep consistency with previous works in the multimedia phylogeny literature. To reconstruct phylogeny trees, previous approaches in multimedia phylogeny follow a 2-step pipeline, by first calculating an *n* × *n* dissimilarity matrix **M** relating all documents. Subsequently, these values are used as input to a tree reconstruction algorithm, using either heuristic-based [3] or optimum branching solutions [4]. The resulting phylogeny tree is represented by a directed acyclic graph, in which every node represents one document, and whose edges indicate the ancestor-descendant relationship. The edge weights are obtained from **M**, whose values are calculated from a dissimilarity function $d(D_{i},D_{j})$ between any two documents $D_{i}$ and $D_{j}$. Roughly speaking, *d* calculates the best mapping of $D_{i}$ onto $D_{j}$’s domain, according to a family of transformations T (in image phylogeny, for instance, it can include resampling, cropping, affine transformation, brightness and contrast change, gamma correction, and compression). Subsequently, the comparison between $D_{i}$ and $D_{j}$ is performed by some point-wise comparison method (e.g., sum of squared differences) [3]. As a result, this function yields small values for similar objects and large values for more distinct objects, that is, the ones that underwent more significant transformations. The choice of such a function is paramount, as every decision made by the subsequent tree reconstruction algorithm is based on these values.

Up to this point, approaches developed in multimedia phylogeny work with a dissimilarity function in a non-metric space, due to the type of transformations evaluated. In image and video phylogeny, $d(D_{i},D_{j})$ does not obey the symmetry and triangle-inequality properties: an image that has undergone cropping or lossy compression is probably descendant from one that has not, for instance. Therefore, $d\left(D_{i},D_{j}\right)\neq d\left(D_{j},D_{i}\right)$, which results in an asymmetric dissimilarity matrix [3]. Nonetheless, this proved to be of fundamental importance for the reconstruction algorithms, as it helped to determine the direction of the modifications (parent-child relationship).

In general, if we consider text documents, common edit operations in text documents (e.g., word exchange, insertion and removal of misspellings, adjectives, adverbs, and even full sentences) are, at first analysis, symmetric, and the functions to calculate their dissimilarity form a metric space. To break this symmetry, some operations or textual features can be given more weight than others. However, it is not always clear which features will give us the strongest clues. Furthermore, assumptions made for a specific scenario are unlikely to work for others, since the directionality in text phylogeny is often dataset dependent, related to the underlying evolutionary process of each set of documents. Therefore, a specific dissimilarity function with unique parameters may be required for each case, being a costly and unreliable endeavor. Thus, in this work, unlike previous approaches in multimedia phylogeny, we propose a framework that explores symmetric dissimilarity functions. In this case, since the cost to map $D_{i}$ onto $D_{j}$’s domain is the same as the one to map $D_{j}$ onto $D_{i}$’s domain, it is not easy to infer which document is the original and which is the modified one solely from the entries of the similarity matrix **M**. Hence, in addition to exploring symmetric dissimilarity functions, we also employ various strategies to infer the root and the direction of relations in a tree of documents, which gives us the following benefits:

- The use of a wider range of dissimilarity functions which are symmetric and do not take edit operations into account (e.g. cosine similarity).
- The problem of finding relationships between documents is uncoupled from solving directionality. Therefore, we can build an undirected graph which depends solely on the entries of **M**, which is a valuable information when orientation is not an issue.
- Several ideas can be seamlessly combined, as we can quickly adopt widely used dissimilarity functions and heuristics into our framework, rather than building a specific method for each case.

**Fig 2:**
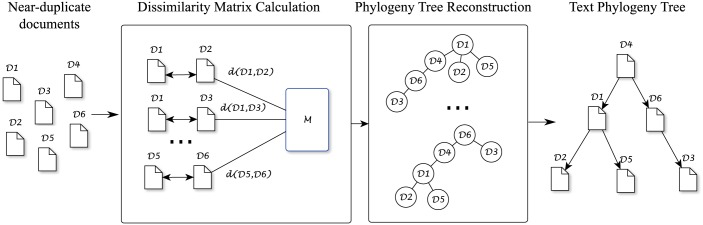
Proposed framework. Given *n* near-duplicate documents, an *n* × *n* symmetric dissimilarity matrix **M** is calculated. This matrix is used to construct an undirected tree using a minimal spanning tree algorithm, from which we can use an heuristic or other strategies to infer the root and the edges’ directions, obtaining the final configuration of the tree.

In Fig 2, we present an overview of the proposed framework. Although it follows the same 2-step pipeline discussed earlier, the algorithms within each of the steps can be custom-tailored according to the particularities of each dataset.

### Dissimilarity Matrix calculation

Given a set of *k* near-duplicate documents, the first task for creating their phylogeny tree consists in calculating their pairwise dissimilarity. In the proposed framework, we have considered three different approaches as detailed below.

#### Edit Distance

For simplicity and efficiency reasons, the choice for such a function is the approach proposed by Wu et al. [33], which is highly efficient when the sequences compared are similar. In this work, we use this algorithm to compare sequences of tokenized words rather than sequences of characters. The function outputs a score, in which a low score denotes similar strings. If one of the input documents is much longer than the other (e.g., a short excerpt of an article compared to the article itself), a high score may be observed, even if the excerpt aligns perfectly with a passage of the article, due to insertions and deletions. On the other hand, if the article is compared to another with the same subject (hence they are similar, but not as much as the previous case), and similar length, a low score may be observed. To compensate for this effect, we added a normalization step, consisting of dividing the unnormalized score by the length of the longer document. For completeness and comparison reasons, we consider both, normalized and unnormalized variants, in the performed experiments.

#### Normalized Compression Distance (NCD)

The second approach uses the Normalized Compression Distance (NCD), a function commonly used for clustering and data mining. It is a function that takes into account the variations in the pairwise compressed sizes of two files *f*_1_ and *f*_2_, namely *C*(*f*_1_) and *C*(*f*_2_), and the compressed size of their concatenation, *C*(*f*_1_ *f*_2_). It can be calculated using the equation below [34]:

$$\begin{array}{c} NCD\left(f_{1},f_{2}\right)=\frac{C\left(f_{1}f_{2}\right)-min\left\{C\left(f_{1}\right),C\left(f_{2}\right)\right\}}{max\{C\left(f_{1}\right),C\left(f_{2}\right)\}}. \end{array}$$ (1)

The distance between two files is measured by comparing the sum of the sizes of *C*(*f*_1_) and *C*(*f*_2_) separately, to their compressed concatenation *C*(*f*_1_ *f*_2_). The returned values represent how different two files are, in which smaller values indicate more similarity between *f*_1_ and *f*_2_. It lies in the interval 0 ≤ *NCD*(*f*_1_ *f*_2_) ≤ 1 + *ϵ*, where *ϵ* is due to imperfection in the compression techniques (in general, it is smaller than 0.1). Common compression algorithms used in this approach include *gzip, bzip2*, and *PPM (Predicition by Partial Matching)*. A further analysis regarding the precision of the distance and the size of the objects for each of them can be found in the paper by Cebrian et al. [35].

#### Cosine Similarity and Term Frequency-Inverse Document Frequency (tf-idf)

The third and last approach combines a convenient way to express textual information in a vector space with a simple and efficient method to compare vectors. First, we extract textual features and compute their weights using a well known technique for text mining, Term Frequency-Inverse Document Frequency (tf-idf), representing each of the documents in the analyzed set as a vector. Then, we compute the similarities between the documents by performing a dot product between the documents’ generated vector representations. Since tf-idf produces normalized vectors, this is equivalent to compute their cosine similarities.

In our framework, we perform this task in three distinct steps: (a) construction of a term dictionary, (b) computation of term weights and (c) calculation of similarities. It is worthwhile to note that steps (a) and (b) together represent a full feature extraction framework. We build the dictionary by aggregating all the terms present in a particular corpus. We can identify different terms according to two parameters: the number of n-grams *n*, and the pre-processing functions P, which are applied to the text before extracting the terms. In our framework, P can assume values in the set {stemming,stopword removal} while *n* can be a subset of {1, 2, 3, 4, 5}. In Table 1 we provide an example for a corpus of a single document with *n* ∈ {1, 2} and P∈{stemming,stopword removal}. In our experiments, we observed the framework performance in various settings of P and *n*. For cosine similarity purposes, the best setting obtained was P=∅ and *n* ∈ {1, 2, 3}.

**Table 1:** Example of feature extraction on a corpus of a single document for *n* ∈ {1, 2} and P∈{stemming,stopword removal}.

**Corpus**	[“The quick brown fox jumps over the lazy dog”]
**Word tokens**	“The”, “quick”, “brown”, “fox”, “jumps”, “over”, “the”, “lazy”, “dog”
**P={stemming}**	“The”, “quick”, “brown”, “fox”, “jump”, “over”, “the”, “lazi”, “dog”
**P={stopword removal}**	“quick”, “brown”, “fox”, “jump”, “lazi”, “dog”
***n* = 1**	“quick”, “brown”, “fox”, “jump”, “lazi”, “dog”
***n* = 2**	“quick brown”, “brown fox”, “fox jump”, “jump lazi”, “lazi dog”
**Final dictionary**	“quick”, “brown”, “fox”, “jump”, “lazi”, “dog”, “quick brown”, “brown fox”, “fox jump”, “jump lazi”, “lazi dog”

Each text document can be represented by a *m*-dimensional vector, where *m* is the number of terms in the dictionary. To effectively compute the term weights, we used Term Frequency-Inverse Document Frequency (tf-idf) [36, 37]. The value of each term increases with the number of times it appears in the document, but it is normalized by its frequency in the corpus. The term frequency-inverse document frequency, or tf-idf(*t*, *f*) score, for a particular term *t* in a document *f* is obtained through the product of two other measures:

$$\begin{array}{c} \text{tf-idf}(t,f)=\text{tf}(t,f)\times \text{idf}(t). \end{array}$$ (2)

In the equation above, tf(*t*, *f*) is the *term frequency*, which counts the number of times *t* occurs in *f*. The second term, idf(*t*), is the *inverse document frequency*, and it provides the information about how frequent *t* is across all documents in the corpus *D* = {*f*_1_, *f*_2_, …, *f*_*n*_}. In this case, frequent terms are likely to have low idf, while rare terms have high idf. By combining these two definitions, we can assign to *t* a weight that is high when *t* occurs many times within a small number of documents, and low when the term appears fewer times in *f*, or appears in many documents [36].

After computing the tf-idf weights, we produce a *k* × *m* feature matrix **F**, where *k* is the number of documents in the corpus (vector space representations), and *m* is the number of terms in the dictionary. We calculate the similarities between the documents by performing dot products between the rows of the feature matrix, producing a *k* × *k* matrix $S=F\times F^{⊺}$. Since we normalize **F** rows while calculating term weights, **S** entries **S**_*ij*_ effectively carry the cosine similarities between documents *i* and *j*. Finally, the dissimilarity matrix **M** is obtained with **M** = |**S** − 1|. For simplicity reasons, we use tf-idf to refer to this similarity calculation in the remaining part of the text.

### Phylogeny Tree Reconstruction

Given the dissimilarity matrix **M**, the proposed approach to reconstruct text phylogeny trees (TPTs) starts by first building a non-oriented tree, since we still cannot infer the directionality of the edges with a symmetric pairwise dissimilarity. This tree can be obtained with a minimum spanning tree algorithm on **M**, such as Kruskal’s algorithm [38]. In this case, the goal is to construct a tree in which the total weight of all edges is minimized. The next step consists in finding the root of this tree. This is not a simple task since little can be assumed regarding the directionality of the relationships in a symmetric set. To address this problem, given *k* documents, we devised three methods to infer the root of the tree, as detailed below.

#### Minimum-cost heuristic

Given an *k*-document non-oriented tree, we can build *k* possible oriented tree configurations, by assigning each of its nodes as root. In this approach, we calculate *k* tree costs *C*_*i*_, each one assuming the tree is rooted at node *i*. Each tree cost is determined by the sum of the edge weights of the potential root *i* to the other nodes in the tree, with all nodes being considered once as a potential root. Finally, we choose as root of the tree the node which generated the tree with minimum *C*_*i*_. When two nodes equally generate trees of minimal cost, we randomly select one of them to be the root of the reconstructed tree.

**Fig 3:**
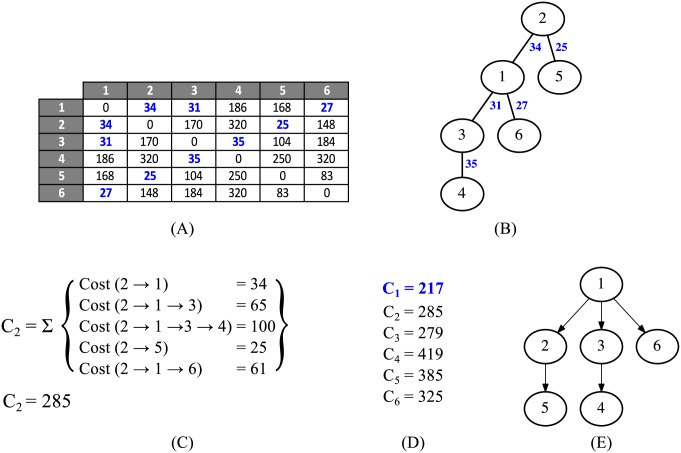
Overview of the Text Phylogeny Tree reconstruction. (A) Given *k* documents, an *k* × *k* symmetric dissimilarity matrix **M** is calculated. (B) An undirected tree with six nodes is constructed, each node representing one of the documents, with the edge weights (dissimilarity values) highlighted in blue. (C) Calculation of the total cost of the tree assuming node 2 as the root. (D) This calculation is performed *k* times, each time considering one of the nodes as potential root. (E) Since *C*_1_ returned the lowest cost, the chosen tree configuration considers node 1 as the root.

In Fig 3, we illustrate a tree reconstruction using this approach. From the dissimilarity matrix **M** relating *k* documents in Fig 3(A), we build the undirected tree using a minimum spanning tree algorithm, as depicted in Fig 3(B). To infer the root of this tree, we calculate the cost of all *k* potential trees, considering each one of the nodes as the root at a time. For instance, to calculate the cost *C*_2_ of the tree assuming node 2 is the root, we calculate the sum of the weights from node 2 to all other nodes as shown in Fig 3(C). Thus, the total cost for this tree is *C*_2_ = 285. The same procedure is repeated for all nodes, obtaining the results shown in Fig 3(D). Since the cost *C*_1_ is the lowest among all calculated costs, then node 1 is chosen as the root of the tree, and the phylogeny tree is depicted in Fig 3(E).

One drawback of this approach is the fact that it works under the assumption that the process of text reuse creates trees that tend to be balanced over time, with respect to the dissimilarities between the nodes. However, if the history of editions of a given document does not create any branchings, for instance, this approach tends to fail, as a tree that has no branchings will have higher cost than the one with branchings, leading the algorithm to choose the wrong reconstruction. As an attempt to broaden the application scope, the approaches we discuss next try to infer the root based on the documents’ content, in a supervised machine learning setting. For these cases, we used two different classification algorithms, which are widely used in the literature: support vector machines and random forests.

#### Support Vector Machines (SVMs)

Support Vector Machines have been extensively used in different research problems, due to its robustness and efficiency in classification tasks [39–41]. In this paper, we employ this algorithm in a supervised machine learning setting, where we aim at classifying a given document as being either **original** or **reused**. For this purpose, it is also worthwhile to represent each document in a vector space. In this case, we use the feature extraction framework defined for the cosine similarity calculation, and the vocabulary was built considering the feature extraction parameters *n* = 1 and P∈{stemming,stopword removal}, after tuning. For term weighing, tf-idf was used. From each training set tree, one positive example (the root) and two negative examples (randomly selected reused documents) are used. Therefore, for a training set consisting of *k* trees and a dictionary of *m* terms, we build a 3*k* × *m* design matrix to be used as input in the algorithm.

#### Random Forests

Random Forests are essentially a combination of decision tree classifiers fitted on random sub-samples of the training data, which was shown to reduce overfitting and increase robustness in various settings [42–44]. Recently, random forests were employed in high dimensional, low-sample size problems similar to our own, yielding results that favorably compare to SVMs [45]. Following this trend, we use this algorithm as another root inferring method. Feature extraction, pre-processing, and training are carried out the same way as described for support vector machines.

## Experimental setup

In this section, we present the methodology used to evaluate the proposed framework. The experiments include a quantitative evaluation in synthetic and non-synthetic datasets (differing by the way the documents were modified: automatically or by Internet users, respectively), and a qualitative evaluation in plagiarism and stemmatology datasets.

### Datasets

For the quantitative evaluation of our approaches, we used two datasets: (a) a synthetic dataset, in which the near-duplicate documents are created according to a prearranged set of parameters, and (b) a real-world dataset comprising text documents collected from *Wikipedia* Featured Articles [46], whose content editions were made by Internet users. Additionally, training and test sets were built from the main datasets, to be used in the decision-making algorithms, SVMs and Random Forests. To allow reproducibility of all experiments, all datasets and the framework source-code will be freely available upon acceptance of this paper in a public repository at http://repo.recod.ic.unicamp.br/public/projects.

#### Synthetic dataset

To construct the synthetic test cases, we used a subset of the *Reuters_50_50* training dataset [47], collecting only articles whose length varies from 350 to 700 words (2,073 documents in total), for efficiency reasons. To build one synthetic evolutionary tree, we randomly choose a document $D_{0}$ from the subset to be its root, and assemble the associated topology using a randomized algorithm. The nodes $D_{i}$ of the tree are modified versions of $D_{0}$, following a set of transformations T given by the topology. Each $D_{i}$ can be modified up to an editing limit *L* to create a new descendant.

The transformations T are functions that perform a sequence of edit operations in a document, such that the overall meaning of $D_{0}$ must be maintained through its descendants. To apply such modifications, we built a set $O_{p}$ composed by four text edit operations, which are highly present in obfuscated plagiarism cases:

- **Synonym exchange:** A given word is replaced by a randomly selected synonym. It is achieved by consulting the WordNet [48] dictionary.
- **Insertion and removal of misspellings:** A correctly spelled word in the text is replaced by a misspelling, or vice-versa. We used two misspellings datasets: the Birkbeck Common Misspellings [49], and the *Wikipedia* Common Misspellings [50].
- **Insertion and removal of modifiers (adjectives and adverbs):** Given a noun or a verb in the text, a modifier is inserted before it, or an existing one is removed. They were added by consulting a subset of the n-gram dataset of the Corpus of Contemporary American English (COCA) [51], and removed by checking their part-of-speech tags using the NLTK package [52].
- **Insertion and removal of sentences:** To perform these transformations, some sentences of $D_{0}$ are held-out before the tree generation. Insertion is handled by selecting a random sentence from this held-out set and returning it to the document. Removal of sentences is also random. Once a sentence is removed, it cannot not be inserted again. To preserve the meaning, the order in which the sentences appear is maintained.

To effectively track the documents’ sentences and words while they are being modified, we represent a given document $D_{i}$ as a nested list of sentence and word tokens $Tokens(D_{i})$, built by first dividing the document into sentences (sentence tokenization) and then dividing each sentence into words (word tokenization). An example follows in Table 2. Each word can be accessed by a pair of keys [*s*_*j*_, *w*_*j*_], which represent the sentence and word indexes, respectively. A word operation (e.g. synonym exchange) is defined as a transformation of only one token $Tokens\left(D_{i}\right)\left[s_{j},w_{j}\right]$. A sentence operation (e.g. removal of sentences) transforms all word tokens which are under the given sentence $Tokens\left(D_{i}\right)\left[s_{j},•\right]=Tokens\left(D_{i}\right)\left[s_{j},w_{0}\right],Tokens\left(D_{i}\right)\left[s_{j},w_{1}\right],...,Tokens\left(D_{i}\right)\left[s_{j},w_{n}\right]$. Any change made to a token (substitution, removal or insertion) counts as a single editing operation.

**Table 2:** Example of textual representation as a nested list of sentence and word tokens.

**Text**	Good muffins cost $3.88 in New York. Please buy me two of them. Thanks.														
**Sentences**	**1**								**2**						**3**
	Good muffins cost $3.88 in New York								Please buy me two of them						Thanks
**Words**	**[1, 1]**	**[1, 2]**	**[1, 3]**	**[1, 4]**	**[1, 5]**	**[1, 6]**	**[1, 7]**	**[1, 8]**	**[2, 1]**	**[2, 2]**	**[2, 3]**	**[2, 4]**	**[2, 5]**	**[2, 6]**	**[3, 1]**
	Good	muffins	cost	$	3.88	in	New	York	Please	buy	me	two	of	them	Thanks

As described in Algorithm 1, first we randomly choose one word or sentence token of $Tokens(D_{i})$ to be transformed by one of the text edit operations in $O_{p}$ at a time and with no replacement, executing it until reaching a number of editing operations $N_{O_{p}}$ previously defined. The number of editing operations $N_{O_{p}}$ is controlled by an editing limit *L*, defined as the proportion of editing operations over the length (in words) of the document. Thus, for instance, for a document with 200 word tokens and *L* = 50%, we perform 100 different word token substitutions, removals or insertions. At each iteration of Algorithm 1, one operation of the set $O_{p}$ is chosen, modifying only one word or sentence. When modifying words, we always add one to the editing operation counter, regardless of the operation type. When modifying sentences, we add the length in words of the sentence to the editing operation counter. To simulate sentence insertion, it is necessary to cache some sentences from the original text document, so when this operation is chosen, we have some sentences available to keep the text coherence. For this case, the proportion of held out sentences chosen was 20%. Furthermore, each operation is chosen with probability relative to some weights, determined through empiric experiments. In our setup, a higher priority was given to synonym exchange, although some variety can be observed since we employed a randomized algorithm.

Following the aforementioned protocol, we built a dataset with two test cases:

- **Progressive editing limit**, in which the same editing limit *L* was enforced to generate all synthetic documents within a tree. *L* varies in the interval *I* = {5%, 10%, …, 50%}, in steps of 5%, so that the trees are composed of either mildly or heavily modified documents. It comprises 1,000 trees generated for each editing limit step, with five 200-element subsets of trees having size |*T*| equal to 10, 20, 30, 40 or 50 elements. In total, 10,000 trees were built, corresponding to 300,000 distinct synthetic documents.
- **Mixed editing limit**, such that *L* is randomly chosen in the interval *I*. The trees can be composed of both, mildly and heavily, modified documents. It comprises 5,000 trees, equally divided among trees having 10, 20, 30, 40, and 50 nodes, corresponding to 150,000 distinct synthetic documents.

With these two sets of test cases, we can analyze how the proposed approaches perform with respect to the size of phylogeny trees, the degree of modifications, and transformation homogeneity (if all objects of a given tree underwent the same degree of transformation, or if some of them were more modified than the others).

**Algorithm 1** Creating the Synthetic Dataset

**Input:** The parent document $D_{p}$, a set of parameters *p*_*word*_ = {*ss*, *ti*, *tr*, *mi*, *mr*} and *p*_*sentence*_ = {*si*, *sr*} used to determine the relative probability of choosing each operation to be applied in words or sentences, respectively (where *ss* stands for synonym substitution, *ti* for typo insertion, *tr* for typo removal, *mi* for modifier insertion, *mr* for modifier removal, *si* for sentence insertion, and *sr* for sentence removal, each of them with a weight value), and the editing limit *L*.

**Output:** The child document *D*_*c*_

1: *editCounter* ← 0      ▹ Initialize editing operations counter

2: *s*_*k*_ ← 0      ▹ Initialize index to retrieve a sentence after tokenization

3: *w*_*k*_ ← 0      ▹ Initialize index to retrieve a word after tokenization

4: $Tokens\left(D_{c}\right)\leftarrow Tokens\left(D_{p}\right)$      ▹ Initialize child document with the parent’s tokens

5: $N_{O_{p}}\leftarrow L\times length\left(Tokens\left(D_{p}\right)\right)$      ▹ Maximum number of editing operations

6: **while** $editCounter<N_{O_{p}}$ **do**      ▹ While the editing limit is not reached

7:  *op* ← *choose*_*operation*(*p*_*word*_, *p*_*sentence*_)      ▹ Choose the operation to be performed according to the weights on the sets *p*_*word*_ and *p*_*sentence*_

8:  **If** *op* ∈ {*ss*, *ti*, *tr*, *mi*, *mr*} **then**

9:   $\left[s_{k},w_{k}\right]\leftarrow random\_word\_index\left(Tokens\left(D_{c}\right)\right)$      ▹ Choose randomly among the pairs of keys pointing to words

10:   $Tokens\left(D_{c}\right)\left[s_{k},w_{k}\right]\leftarrow apply\_word\_operation\left(op,Tokens\left(D_{c}\right)\left[s_{k},w_{k}\right]\right)$      ▹ modifying word on position [*s*_*k*_, *w*_*k*_]

11:   **If** the operation is successful **then**      ▹ Some words may not have synonyms or modifiers, for instance. When the operation is unsuccessful, $Tokens\left(D_{c}\right)\left[s_{k},w_{k}\right]$ does not change

12:    *editCounter* ← *editCounter*+1

13:   **end if**

14:  **else if** *op* ∈ {*si*, *sr*} **then**

15:   $s_{k}\leftarrow random\_sentence\_index\left(Tokens\left(D_{c}\right)\right)$      ▹ Choose randomly among sentence indexes

16:   $Tokens\left(D_{c}\right)\left[s_{k},•\right]\leftarrow apply\_sentence\_operation\left(op,Tokens\left(D_{c}\right)\left[s_{k},•\right]\right)$      ▹ Modifying all tokens under sentence *s*_*k*_. In our case, sentence operations can either return an empty list of word tokens or a new list of word tokens taken from the held out sentence set

17:   **if** the operation is successful **then**

18:    $editCounter\leftarrow editCounter+length(Tokens\left(D_{c}\right)\left[s_{k},•\right])$      ▹ For operations in sentences, the total count of editing operations is equal to the length (in words) of the sentence.

19:   **end if**

20:  **end if**

21: **end while**

22: $D_{c}\leftarrow to\_text\left(Tokens\left(D_{c}\right)\right)$      ▹ Collapsing the nested list of tokens back to a single string

23: **return** *D*_*c*_

#### Real-world dataset

In this dataset, we used the page histories of several featured articles from *Wikipedia*. Each page history shows the order in which changes were made to any editable page, and the difference between any two versions. They were obtained using *Wikipedia*’s export tool [53]. The data is exported in the form of xml dumps, from which plain text was extracted using a parsing tool [54]. After this cleaning process, we obtained 859 page histories, with up to 1,000 revisions each.

Different from the previous dataset, the phylogeny trees in *Wikipedia* have one particularity: in their original format, they are graphs with no branches. This happens because the article revisions are always recorded linearly, with each new revision being placed as a descendant of the previous one, without branchings. However, with respect to the document contents, this is not always true. Since we only considered changes to the articles’ actual content (changes to references, table of contents, or images, for instance, were discarded), many revisions within the edit history were actually the same. In addition, *Wikipedia* is known for its collaborative (or conflictive) nature, so the presence of edit wars and revision reverts [55, 56] are noteworthy.

**Fig 4:**
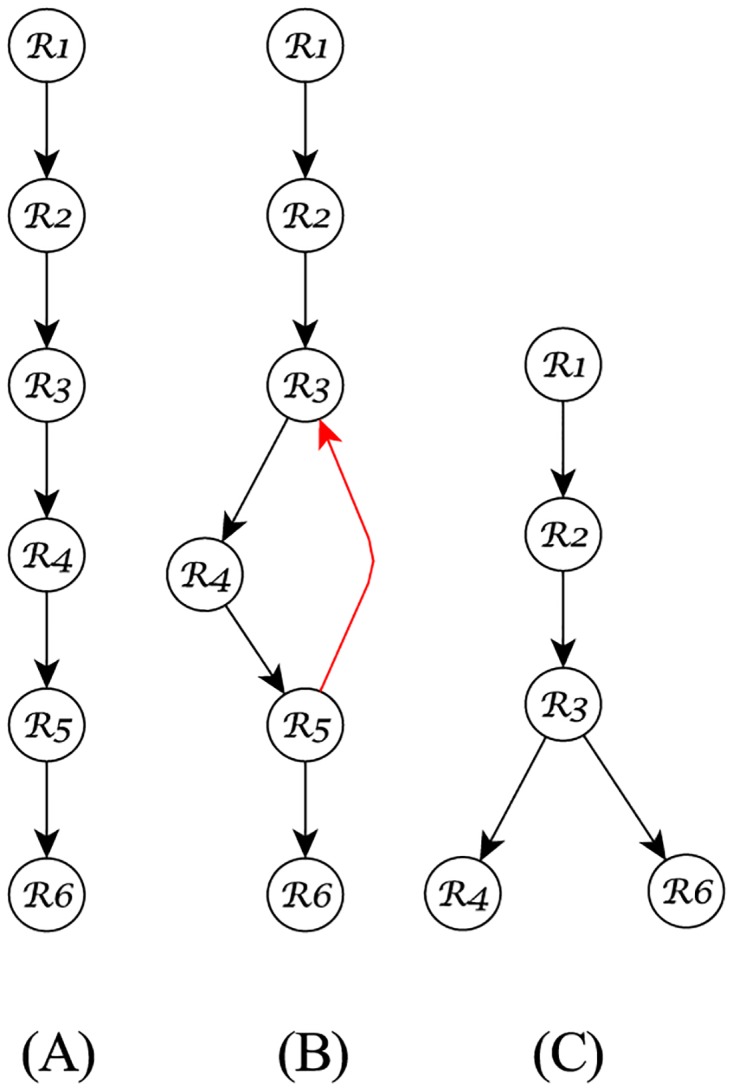
Revision reverts in *Wikipedia* dataset. (A) A raw revision tree, as originally obtained from *Wikipedia*, without branchings. (B) In some cases, one revision reverts to an existent one. In this case, $R_{5}$ reverts to $R_{3}$. (C) A new branching is created in $R_{3}$, while node $R_{5}$ is discarded.

**Fig 5:**
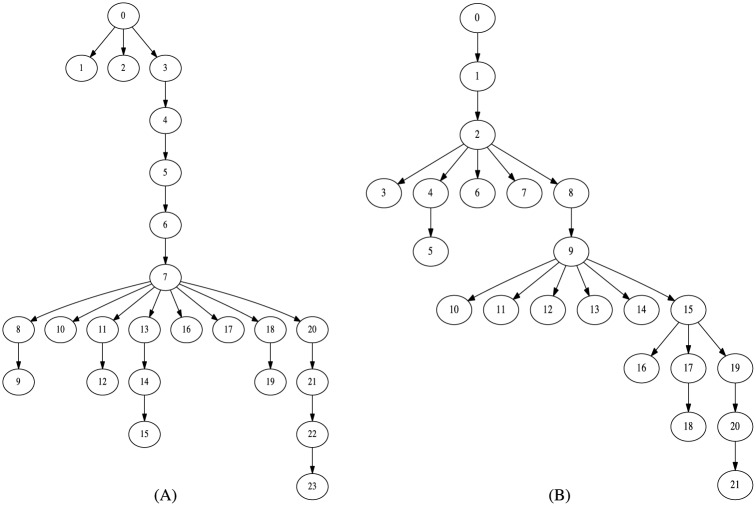
First instances of the processed revision histories of two *Wikipedia* articles. (A) “Partners in Crime (Doctor Who)”. (B) “Convention of 1833”.

Revision reverts are particularly relevant, as they can create branches in the degenerate tree structures. For instance, if a given revision $R_{k}$ was reverted to $R_{l}$, it should be discarded, and $R_{k+1}$ should be linked to $R_{l}$ (unless it is also equal to another revision) and the subsequent documents would be part of this new branch. Therefore, to build a reliable text phylogeny dataset from *Wikipedia* data, we devised an algorithm that deals with the aforementioned aspects (Algorithm 2). It takes a raw revision tree and transforms it by removing identical instances and creating branchings where reversions exist, as shown in Fig 4. Most of the revision histories remained without branches (or with minor branches) after being processed by this algorithm, but interesting patterns could be observed for some cases. We show some interesting examples in Fig 5.

Similarly to the previous dataset, trees were set to have size |*T*| ∈ {10, 20, 30, 40, 50}, starting from the first revision. The set comprises a total of 4,289 trees, as some revision histories do not have at least 50 unique documents, and were, therefore, discarded. It is important to notice that in this dataset, the number of linear trees is directly related to the number of nodes in it, since the higher the number of nodes, higher are the chances that there is a reversion, and therefore, a branching in the tree. In our dataset, for trees with 10 nodes, for instance, approximately 73.74% of the trees are linear; for trees with 20 nodes, this number decreases to 54.60%, and this number keeps decreasing for a higher number of nodes, increasing the complexity of the dataset. More details regarding the analysis of the dataset is included in the supporting information along with the dataset’s description (Tables A-D in S1 File).

**Algorithm 2** Creating the ground-truth for the Wikipedia dataset

1: *N* ← number of revisions

2: *R*[*k*]← revision number *k*, where *k* = 0, 1, 2, …, *N* − 1

3:**for** *i* from 1 to *N* **do**

4:  *j* ← *i* − 1

5:  *R*[*i*] is a descendant of *R*[*i* − 1]

6:  **while** (*R*[*i*] exists and *j* > 0) **do**

7:   **if** *R*[*i*] is equal to *R*[*j*] **then**

8:    discard *R*[*i*]

9:    *R*[*i*+1] is a descendant of *R*[*j*]

10:   **end if**

11:   *j* ← *j* − 1

12:  **end while**

13: **end for**

#### Training and test sets for the supervised machine learning experiment

To perform experiments with SVMs and Random Forests, the main datasets were divided between training and test sets. As many trees in the main datasets share the same original document (the synthetic set is composed of 15,000 trees, but built from 2,073 base documents, and the real-world dataset comprises 4,289 trees built from 859 revision histories), we randomly selected test and training examples that do not share the same base document. We considered a training and test set proportion of 50%. Therefore, for the synthetic case, we built a training set of 1,037 examples and a test set of 1,036 examples. For the real-world dataset, the number of examples of the training and test set, were, respectively, 430 and 429 trees.

As previously mentioned in the Dissimilarity Matrix Calculation section, feature extraction is performed by first creating a term dictionary, which depends on the number of n-grams extracted *n* and the pre-processing operations applied to the text P. For the supervised machine learning algorithms, feature extraction was performed with parameter settings *n* = 1 and P∈{stemming,stopword removal} for both, SVM and Random Forests, approaches.

In addition, we carried out simple hyperparameter tuning, experimenting with the number of estimators and the information criterion on Random Forests, obtaining the best results with *n*_*estimators*_ = 500 and criterion=information gain. For SVMs, we tried linear and RBF kernels, with the former presenting the best computational cost-benefit. Finally, we trained and tested our models with the best feature extraction and algorithm parameters. The algorithms were implemented with scikit-learn[57], a machine-learning Python framework.

### Evaluation metrics

To assess the performance of the proposed framework, we use the same evaluation metrics used in previous work of multimedia phylogeny [3], which are calculated with respect to the correct (original) phylogeny tree *T*_1_, and a reconstructed phylogeny tree *T*_2_. These metrics have complementary properties that provide an overview on the reconstruction algorithm’s behavior, being defined as follows:

- **Root**: It compares the reconstructed root *R*_2_ with the ground-truth root *R*_1_. With this metric, we can evaluate how good the proposed method is to correctly retrieve the original (source) document from a set of near-duplicate documents. In documents plagiarism, for instance, correct identification of the root helps to determine who wrote the original document within the set under analysis.
  $$\begin{array}{c} Root\left(T_{1},T_{2}\right)=\{\begin{array}{cc} 1, & \text{if}\;Root\left(T_{1}\right)=Root\left(T_{2}\right).\\ 0, & \text{otherwise}. \end{array} \end{array}$$ (3)
- **Directed Edges**: It evaluates the edge connections and their directionality. Therefore, if a directed edge connecting two nodes in *T*_2_ is the same as in *T*_1_, then it is considered correct. Considering *n* documents, with this metric, we can evaluate whether we correctly found the direction of the parent-child relationship between each pair of documents from *T*_2_ with respect to *T*_1_.
  $$\begin{array}{c} Edges\left(T_{1},T_{2}\right)=\frac{Edges\left(T_{1}\right)\cap Edges\left(T_{2}\right)}{n-1} \end{array}$$ (4)
- **Leaves**: The leaves of the tree are the documents without descendants (terminal nodes). This metric evaluates whether the documents on the leaves of *T*_2_ are the same found in *T*_1_.
  $$\begin{array}{c} Leaves\left(T_{1},T_{2}\right)=\frac{Leaves\left(T_{1}\right)\cap Leaves\left(T_{2}\right)}{Leaves\left(T_{1}\right)\cup Leaves\left(T_{2}\right)} \end{array}$$ (5)
- **Ancestry**: For each node, it evaluates whether all its ancestors until the root in *T*_1_ are correctly found in *T*_2_. Thus, we can evaluate the reconstruction with respect to each node parent, grandparent, great-grandparent, and so on.
  $$\begin{array}{c} Ancestry\left(T_{1},T_{2}\right)=\frac{Ancestry\left(T_{1}\right)\cap Ancestry\left(T_{2}\right)}{Ancestry\left(T_{1}\right)\cup Ancestry\left(T_{2}\right)} \end{array}$$ (6)
- **Indirect Edges**: Similarly to the metric Directed Edges, it returns the number of correct edge connections, but disregards directionality. Different from approaches for image and video phylogeny, in text phylogeny, it is harder to determine the direction of the relationship since we have symmetric edge values. Thus, we created this metric for evaluating the non-oriented tree reconstruction.
  $$\begin{array}{c} IndirectEdges\left(T_{1},T_{2}\right)=\frac{IndirectEdges\left(T_{1}\right)\cap IndirectEdges\left(T_{2}\right)}{n-1} \end{array}$$ (7)
- **Depth**: It measures the distance, in number of edges, between the original root *R*_1_ and the reconstructed root *R*_2_. Through the results of this metric, it is possible to evaluate how many edges away the reconstructed root is with respect to the correct root. Considering *dist*(*i*, *j*, *T*) the function that calculates the number of edges that separates two nodes *i* and *j* on the tree *T*, the Depth is calculated as the distance of the roots in the reconstructed tree:
  $$\begin{array}{c} Depth\left(T_{1},T_{2}\right)=dist\left(Root\left(T_{1}\right),Root\left(T_{2}\right),T_{2}\right) \end{array}$$ (8)

### Experiments and Results

In the first part of the experiments, we calculated the dissimilarity among near-duplicate documents for each of the approaches previously explained, with some variation of parameters. For the edit distance, we performed experiments using its unnormalized and normalized version; for the NCD we used the *bzip2* compressor, and for tf-idf, we tested word and character-based *n*-grams separately, and their combinations (*n* = {1, 2, 3, 4, 5}), with best results achieved by the combination of word-based 1-, 2-, 3-grams.

### Minimum-cost heuristic

**Fig 6:**
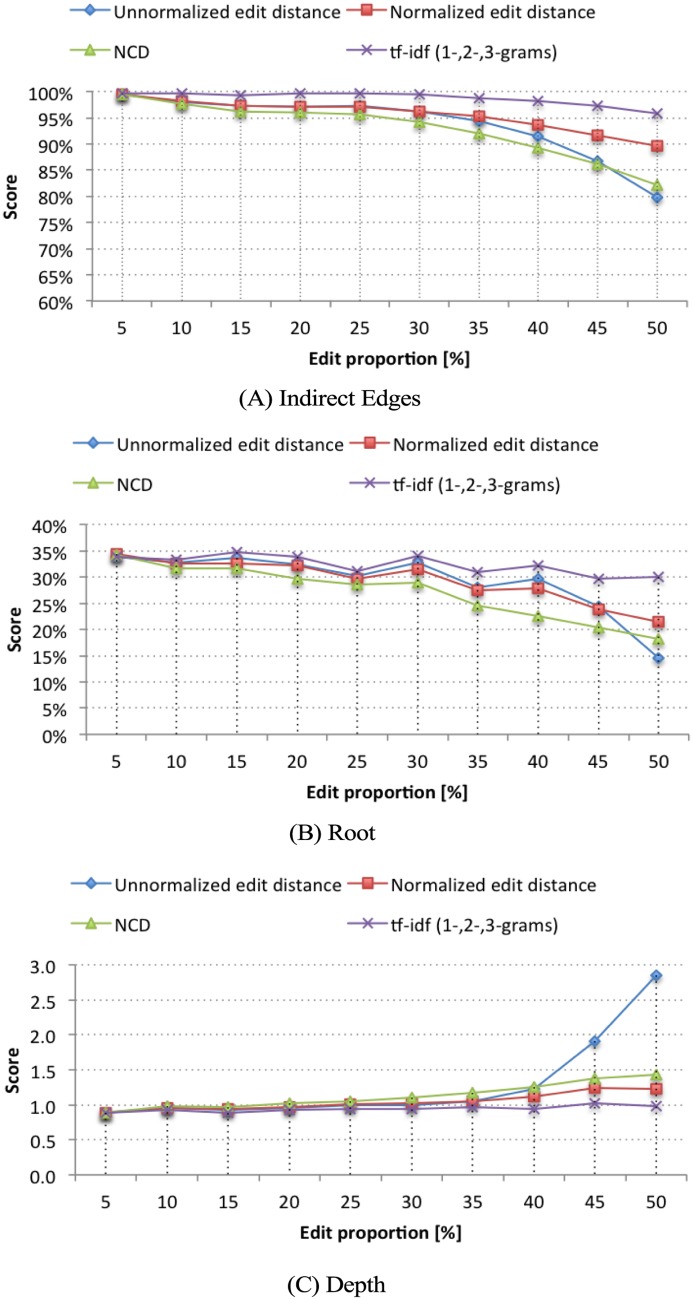
Average of progressive editing limit results. Metrics: (A) Indirect edges, (B) Root, and (c) Depth. The reconstruction using tf-idf (1-, 2-, 3-grams) had the best results, followed by the normalized edit distance.

We start the discussion of the experimental results with a comparison among the performance of the dissimilarity functions, combined with the minimum-cost heuristic. To avoid cluttering the paper, in Fig 6 we show the average results (over tree sizes), for the progressive editing limit case of the synthetic dataset only for three metrics: Indirect Edges, Root, and Depth, since Leaves and Directed Edges metrics had similar behavior to the Indirect Edges, and Ancestry results are closely related to the metric Root. The ommited results can be found in the supporting information (Figs A-B and Tables E-I in S1 File).

Among all approaches, the reconstruction using tf-idf word-based 1-, 2-, 3-grams presented the best results, followed by the normalized edit distance. The unnormalized edit distance presented slightly better results than NCD up to *L* = 35%, when the document length begins to become a relevant issue, as the transformations become more intense. These results also show the importance of normalization: while the remaining normalized methods managed to maintain robustness after reaching *L* = 35%, the unnormalized edit distance results started to rapidly deteriorate. Nonetheless, the reconstruction of a non-oriented tree was nearly perfect for most of the *L*-range for all dissimilarity functions, as evidenced by the Indirect Edges metric (Fig 6(a)).

Regarding directionality, there is still room for improvement, as the minimum-cost heuristic yielded an accuracy of approximately 30% in the Root metric (Fig 6(b)). However, as the Depth metric results show (Fig 6(c)), the reconstructed root is, on average, one edge away of the original root. Therefore, even when we do not find the correct root, we can reduce the problem to a small set of elements with high probability of containing the correct root. This result is also confirmed in Table 3 (complemented by Table E in S1 File), which shows the percentage of times (%) that the reconstructed root or one of its neighbors was the actual root (*neighbor probability*). We only show the results for the two best performing cases (normalized edit distance and tf-idf), and considering *L* = 50%. As an example, for trees with 50 nodes and dissimilarity calculated using tf-idf, we could assert with more than 78% confidence that the original root lay within a 7-element subset close to the reconstructed root. Lastly, Leaves and Directed Edges show promising results, with accuracy over 90% along the entire *L*-range with tf-idf.

**Table 3:** Neighbor probability. Percentage of times the reconstructed root or one of its immediate neighbors was the original root. In this case, *L* = 50% and the dissimilarity metrics are combined with the minimum-cost heuristic.

	Normalized edit distance		tf-idf (1-, 2-, 3-grams)	
|*T*|	neighbor prob. (%)	set size	neighbor prob. (%)	set size
10	76.50	4.61	81.50	4.67
20	68.50	5.19	77.00	5.34
30	70.50	5.67	76.00	5.91
40	64.00	5.86	74.00	6.14
50	64.00	6.08	78.00	6.55

For the synthetic case with mixed editing limit, the results are also promising, showing robustness of the proposed approach when reconstructing trees composed of a non-homogeneous combination of mildly (*L* ≤ 25%) or heavily (25% ≤ *L* ≤ 50%) modified documents. In agreement with the progressive editing limit case, the best results were achieved by tf-idf word-based 1-, 2-, 3-grams. In Table 4, we show the results for both, the progressive (*L* = 50%) and the mixed editing limit, using tf-idf 1-, 2-, 3-grams, according to each tree size |*T*| (Figs A-B, and Tables F-G in S1 File).

**Table 4:** Results for the best performing method (tf-idf 1-, 2-, 3-grams), in the synthetic dataset.

**Progressive editing limit (*L* = 50%)**						
|*T*|	**Ind. Edges (%)**	**Dir. Edges (%)**	**Leaves (%)**	**Root (%)**	**Ancestry (%)**	**Depth**
10	98.00	88.20	95.70	30.50	64.20	0.91
20	96.00	90.90	95.90	33.50	69.40	0.96
30	95.70	92.10	95.60	28.00	70.90	1.03
40	94.80	91.80	94.80	27.50	70.90	1.08
50	94.40	92.20	94.10	30.00	74.10	0.98
**Mixed editing limit**						
|*T*|	**Ind. Edges (%)**	**Dir. Edges (%)**	**Leaves (%)**	**Root (%)**	**Ancestry (%)**	**Depth**
10	97.00	86.90	95.30	31.90	63.70	0.93
20	96.90	91.70	97.00	29.10	68.40	1.00
30	96.60	93.00	97.20	28.30	70.30	1.05
40	96.60	93.70	97.00	26.70	71.30	1.10
50	96.50	94.20	97.10	30.70	73.50	1.06

With respect to the *Wikipedia* dataset, rather than tf-idf, the edit distance variants presented the best results in a close range. This result is not unexpected, since tf-idf cannot capture some subtle modifications which are present in *Wikipedia* revisions, such as changes in the ordering of words. Moreover, *Wikipedia* documents are slowly edited over time. Hence, sudden text length variations are not an issue. On the other hand, tf-idf presented the best results regarding Depth, and therefore could be used as input to the minimum-cost heuristic if we intended to optimize this metric. Since *Wikipedia* dataset is comprised mostly of trees without branchings, and the minimum-cost heuristic searches for the most balanced tree configuration available, all results, especially for the Root and Ancestry metrics, underwent a sharp decrease compared to the ones obtained with the synthetic dataset. However, we were able to obtain an accuracy of around 87% for the Indirect Edges metric, showing that non-oriented trees closely resemble an equivalent graph for the original trees. Thus, although in this case a full automatic reconstruction of a directed tree may be difficult, the non-oriented tree can be reliably used as a starting point to further analysis performed by a forensic expert. Table 5 (Table H in S1 File) shows the results for the unnormalized edit distance, and tf-idf (1-, 2-, 3-grams).

**Table 5:** Results for the minimum cost heuristic-based reconstruction for *Wikipedia* dataset. In this case, the edit distance variants presented the best results, as tf-idf cannot capture some subtle modifications in *Wikipedia*, such as in the ordering of words. In particular, tf-idf presented the best result for Depth metric.

**Unnormalized edit distance**						
|*T*|	**Ind. Edges (%)**	**Dir. Edges (%)**	**Leaves (%)**	**Root (%)**	**Ancestry (%)**	**Depth**
10	87.90	44.90	39.80	0.58	21.60	3.57
20	87.50	45.40	35.30	0.12	19.80	7.10
30	87.50	46.30	34.10	0.70	19.30	10.50
40	87.80	47.30	33.70	0.35	19.30	13.80
50	88.00	47.60	33.70	0.23	19.20	17.20
**tf-idf (1-, 2-, 3-grams)**						
|*T*|	**Ind. Edges (%)**	**Dir. Edges (%)**	**Leaves (%)**	**Root (%)**	**Ancestry (%)**	**Depth**
10	80.50	39.20	32.20	0.12	18.60	3.26
20	78.80	38.80	25.40	0.00	16.80	6.02
30	78.20	38.60	23.20	0.00	15.70	8.67
40	78.40	39.10	22.70	0.00	15.60	11.3
50	78.20	39.10	22.40	0.00	15.40	13.8

### Supervised machine learning algorithms

In the following experiments, we devise some techniques to take advantage of two well-known machine learning algorithms for a better decision-making process for finding the root of the trees as well as the edges’ directions. Our motivation for using Random Forests (RF) along with SVMs was twofold: (a) to show it is valid to use any learning algorithm of the researcher’s choice in our framework; and (b) to compare the performance of RF to SVMs, as previous works showed that RF compared favorably to SVMs in a diverse set of problems and especially when high dimensionality and small training set size conditions are at stake. Therefore, to correctly compare the algorithms and show that the framework can easily accept various learning methods, we provided the same input (training set) for SVMs and RF, and also compared their performance with the same test set.

For both synthetic and real test cases, a term dictionary was built from the training examples by extracting 1-grams after stemming and stopword removal. If some term in the test documents was not present in the term dictionary, it was ignored. Since the documents in training and test sets were similar in style and sometimes content (encyclopedic articles and news articles), this was not common. Therefore, the term dictionary built with the training set documents was suitable for representing test documents as well. For the sake of simplicity, we will only display results that combine the supervised machine learning approaches with the best-performing dissimilarity functions for the proposed test cases: tf-idf for synthetic data, and edit distance (normalized) for Wikipedia data. In our experiments, RBF and linear kernels were used, with the best results achieved by the latter.

Considering SVMs, we greatly improved the directed tree reconstruction in both synthetic and real test cases, as Table 6 shows. In the synthetic case, SVMs achieved near-perfect results in all metrics, with the most remarkable improvement in the Root metric, obtaining an accuracy above 96%, compared to around 30% with the minimum-cost heuristic (Table 4). In this case, the use of positive and negative training samples to extract textual features, allied with a classifier was effective, since the original documents in the synthetic dataset were written by humans (the original authors of the articles), while the near-duplicate documents were edited by a single bot (which may use a limited vocabulary, especially when we consider spelling mistakes). Therefore, the task of finding the root becomes the one of finding which document was written by a human, on which SVMs can perform very well.

**Table 6:** Tree reconstruction using SVMs. Best results were achieved using a linear kernel. The best-performing dissimilarity function in each case was used.

**Synthetic dataset (tf-idf 1-, 2-, 3-grams)**						
|*T*|	**Ind. Edges (%)**	**Dir. Edges (%)**	**Leaves (%)**	**Root (%)**	**Ancestry (%)**	**Depth**
10	98.90	98.60	99.30	97.90	98.20	0.02
20	98.50	98.30	98.50	99.40	97.60	0.01
30	97.90	97.70	98.60	96.50	97.20	0.04
40	98.00	97.80	98.30	98.20	97.30	0.02
50	98.00	97.80	98.20	98.20	97.30	0.02
***Wikipedia* dataset (Unnormalized edit distance)**						
|*T*|	**Ind. Edges (%)**	**Dir. Edges (%)**	**Leaves (%)**	**Root (%)**	**Ancestry (%)**	**Depth**
10	88.40	61.10	50.70	44.20	54.60	2.29
20	85.00	62.20	35.40	47.10	58.10	2.54
30	88.40	68.90	36.20	50.60	64.70	2.63
40	87.70	70.70	37.00	53.60	64.60	2.76
50	88.10	67.90	34.50	38.80	62.20	5.11

Regarding *Wikipedia* data, combined with edit distance, great improvements were also observed, as the previous root inference method failed to work with trees without branchings. The Root metric rose to approximately 45%, while Ancestry increased to approximately 51.50%. Depth was also greatly reduced, especially for large trees. Directed Edges results had an increase of about 20% to 30%, depending on tree size, lying in the 60% to 70% accuracy range. However, despite of also showing substantial increase, Leaves continued to show modest results: as trees have few branchings, there are very few terminal nodes, which increases the challenge of finding them.

Using Random Forests, we had similar performance for the synthetic data, hence, these results are ommited in this paper (Table I in S1 File). On the other hand, we obtained improvements for *Wikipedia* trees reconstruction in comparison to SVMs, as Table 7 shows. The accuracy for the Root metric was above 45% for all tree sizes, along with moderate improvements in Directed Edges, Ancestry and Depth metrics. These results are in accordance with the most recent developments in machine learning, which show that Random Forests outperform SVMs in various settings [45].

**Table 7:** Tree reconstruction using Random Forests (*Wikipedia* dataset). After several tests, we obtained the best results with 500 estimators and information gain as split quality criterion.

*Wikipedia* dataset						
|*T*|	Ind. Edges (%)	Dir. Edges (%)	Leaves (%)	Root (%)	Ancestry (%)	Depth
10	88.40	64.60	54.40	54.70	61.10	1.87
20	85.00	63.40	36.10	47.10	58.10	2.42
30	88.40	69.50	35.70	51.70	65.40	2.46
40	87.70	71.40	36.00	53.60	66.20	2.37
50	88.10	70.10	35.10	45.90	65.00	3.79

To further broaden the scope of our comparison, we resorted to an experiment with increasingly larger trees, composed of |*T*| = {100, 200, 300, 400} nodes, extracted from the *Wikipedia* revision histories. As many of the revision histories do not have that many unique documents, the test set was reduced, being composed of 381 trees in total, of which only 38 have 400 nodes. Therefore, we present this experiment in a more qualitative fashion. Furthermore, it is worth noting that for trees of such size, all dissimilarity functions apart from tf-idf become computationally inefficient. Thus, in this case only, we used tf-idf in conjunction with a machine learning method for dealing with *Wikipedia* data. In this experiment, reconstruction of non-oriented trees remained reliable even in the most challenging cases of trees with 400 nodes, with Indirect Edges accuracy above 80%, as Table 8 shows. Regarding the remaining metrics, accuracy worsened, as expected. However, considering the challenge faced, Root metric showed a surprising accuracy of 28.90% in 400-node trees. Random Forests, similar to the previous case, showed results that compare favorably to the ones obtained with SVMs.

**Table 8:** Results for *Wikipedia* dataset using SVM and Random forests for trees with higher number of nodes. In this particular case, due to the higher order of the trees, rather than using edit distance, we used tf-idf, as this method is more efficient, and made the experiment practical considering time and resources constraints.

**SVM**						
|*T*|	**Ind. Edges (%)**	**Dir. Edges (%)**	**Leaves (%)**	**Root (%)**	**Ancestry (%)**	**Depth**
100	84.50	59.60	25.30	35.40	52.00	7.95
200	83.00	52.80	33.00	20.70	38.80	32.90
300	82.60	54.50	30.00	25.90	40.90	32.40
400	81.70	55.00	33.80	13.20	41.90	40.50
**Random Forests**						
|*T*|	**Ind. Edges (%)**	**Dir. Edges (%)**	**Leaves (%)**	**Root (%)**	**Ancestry (%)**	**Depth**
100	84.50	60.70	26.00	40.90	53.60	7.64
200	83.00	55.90	33.20	27.60	43.50	27.50
300	82.60	54.70	30.10	36.20	42.60	30.80
400	81.70	55.30	34.00	26.30	42.40	38.30

**Fig 7:**
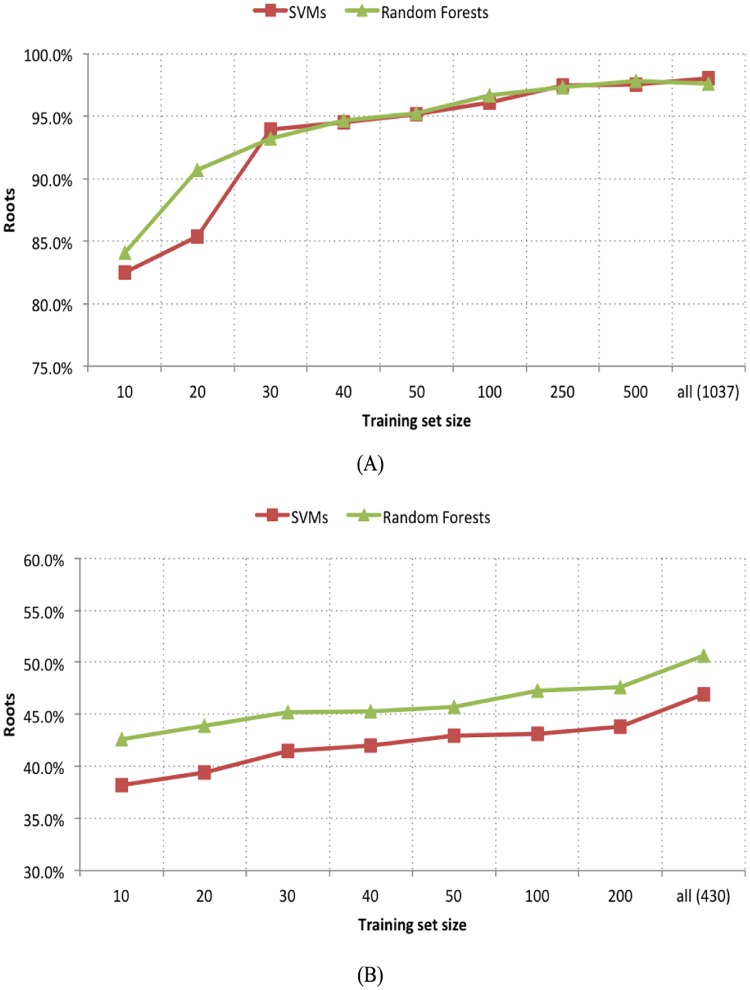
Training effectiveness of SVMs and random forests. In both test cases, with approximately 20 to 30 training trees (60 to 90 unique documents), we were able to achieve a performance that remained comparable to using the full training set.

Finally, let us consider a final experiment regarding SVMs and Random Forests. It consisted of increasingly reducing the size of the training set, to compare the training effectiveness of both algorithms. Fig 7 presents the graphics comparing the results (Root accuracy) for both synthetic and real datasets. In both test cases, with approximately 20 to 30 training trees (60 to 90 unique documents), we were able to achieve a performance that remained comparable to the one using the full training set (1,037 documents for the synthetic dataset, and 430 documents for the *Wikipedia* dataset). Random forests were, in general, slightly more accurate and effective than SVMs, except for close results at some points in the synthetic case.

### Computational time

To present a comparison between the computational time spent by each of the approaches during the dissimilarity computation step, we selected a subset of the synthetic dataset, considering the test case of mixed editing limit. This subset includes 500 different phylogenies, with trees having number of nodes varying between 10 to 50. Table 9 shows the average time spent for each of the approaches. The edit distance is the most computationally expensive, being around 8 times slower than NCD, and approximately 75 times slower than tf-idf. Thus, tf-idf presented the lowest computational time, also being one of the most effective. These experiments were performed in a MacBook Air, Intel Core i7, 2GHz, with 8GB of memory, and running OS X Yosemite 10.10.4.

**Table 9:** Average time for the dissimilarity calculation (seconds).

Edit distance	NCD	tf-idf
15.137	1.538	0.199

### Application in Document Plagiarism

The judgment of whether some document was plagiarized from another one is a very challenging task, and far from being completely solved. For a set of suspicious documents, some of the main challenges include the fact that we do not really know which documents are related, which are just similar, and which ones are the result of real plagiarism. Although plagiarism detection is not the main goal of this work, in this section we provide a qualitative evaluation of our framework by showing how it would perform in this application’s domain.

In general, with the lack of real-case datasets, most of the plagiarism detection methods are evaluated using artificial or simulated datasets (e.g., [30, 58–62]). Although in both cases different degrees of obfuscation are used, in the former, the documents are automatically generated, while in the latter, subjects are asked to create the plagiarised copies. In our experiments, we chose a corpus of *short answers* [59], with simulated cases of plagiarism, as they are closer to what can be observed in real plagiarism.

**Fig 8:**
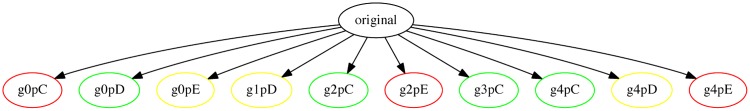
Plagiarism tree example. The labels follow the nomenclature in the dataset, and the colors, the classification given by the dataset [59]: near copy (green), light revision (yellow), and heavy revision (red).

The short answers dataset comprises 100 documents divided in five groups (A-E), each group being one short answer (200-300 words) to a different topic. The answers were written by 19 participants, including native and non-native English speakers, restricted to students with some familiarity with Computer Science. The original documents are articles from *Wikipedia*, and the answers were obtained using four different approaches [59]: (i) near copy (cut-and-paste from any part of the original article), (ii) light revision (answer based on the original article, changing it in some basic ways, without radically changing the order of information found in sentences), (iii) heavy revision (no constraints on how the text was modified, using different words and structure, and splitting or combination of sentences), and (iv) non-plagiarism (lecture notes and textbooks were provided to the participants, and any other material could be used, except *Wikipedia*). Within the 100 documents, there are 5 sources, 57 documents classified as near copy, light revision or heavy revision, and 38 non-plagiarised examples. Fig 8 shows an example of a tree generated by this dataset. The trees have only one level, as there is one original source, and the generated copies do not create new copies. We left the non-plagiarised cases out of this example tree, and the colors in the nodes represent each of the levels in which the documents were plagiarised: near copy (green), light revision (yellow), and heavy revision (red).

**Fig 9:**
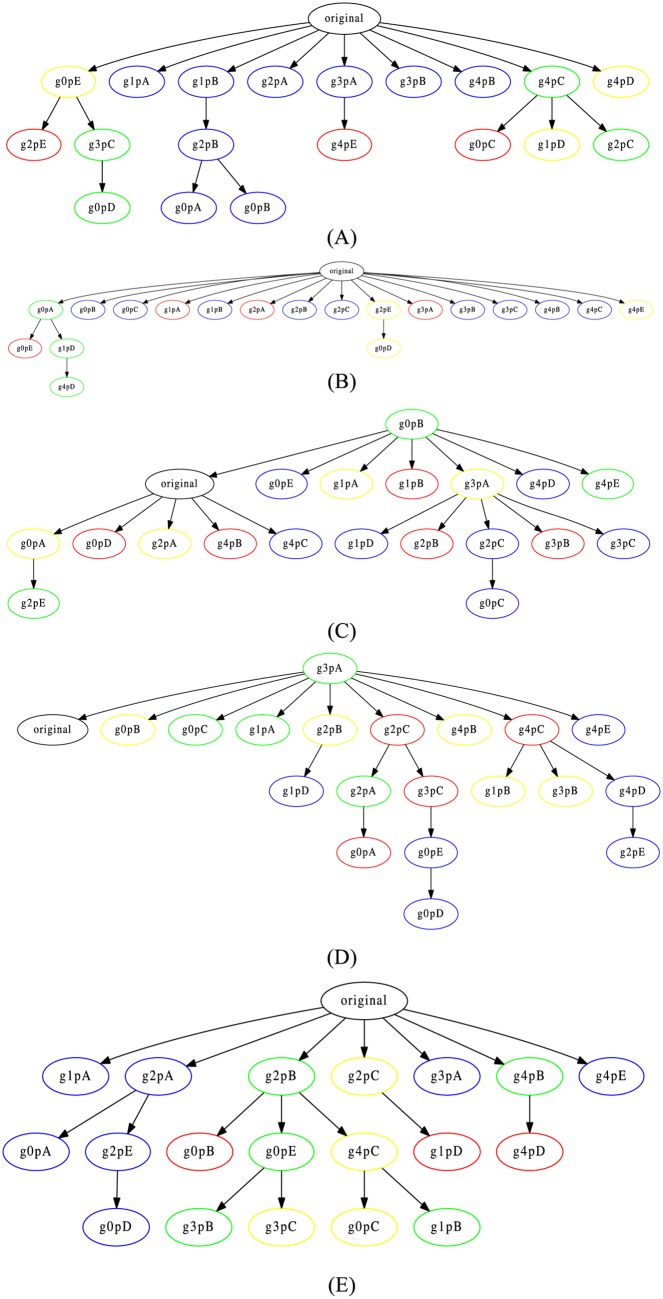
Reconstructed trees in the plagiarism dataset. The trees were reconstructed using tf-idf (1-, 2-, 3-grams), and the minimum cost approach. Each tree (A-E) corresponds to one of the five groups of answers.

In Fig 9, we show the trees of the five documents belonging to this dataset using our proposed framework. The trees were reconstructed using tf-idf (1-, 2-, 3-grams), and the minimum cost approach (Section Materials and Methods). Each tree (A-E) corresponds to one of the five groups of answers. It is important to notice that our framework does not make any selection in the beginning of the algorithm, to separate suspicious cases from non-plagiarism cases. It assumes all documents are related; therefore, the non-plagiarism cases also appear in the reconstructed trees, represented by the nodes in blue. Nonetheless, we were able to identify the original document as root of the tree in three out of five cases, and for the two remaining cases, the original document was placed as an immediate neighbor of the document identified as the root of the tree (Fig 9(C) and 9(D)).

In addition, as expected, documents with heavy revision (red nodes) were all placed as leaves of the tree in the cases which we correctly identified the original document as the root, since they differ the most from the original document. Even though the minimum cost approach works better with balanced trees, we were still able to obtain a tree reconstruction closer to the original tree configuration in Fig 9(B).

Although we carried out this experiment with a small dataset, it gave us an insight of which points in the framework should be changed to be applied in plagiarism cases. For instance, it would be interesting to implement a method in which from a large pool of documents we extract which documents will be used for generating the tree. Our current framework assumes the documents are all somehow related, and evaluation of the reconstructed tree for these cases would probably require the aid of a plagiarism expert.

### Application in Stemmatology

**Fig 10:**
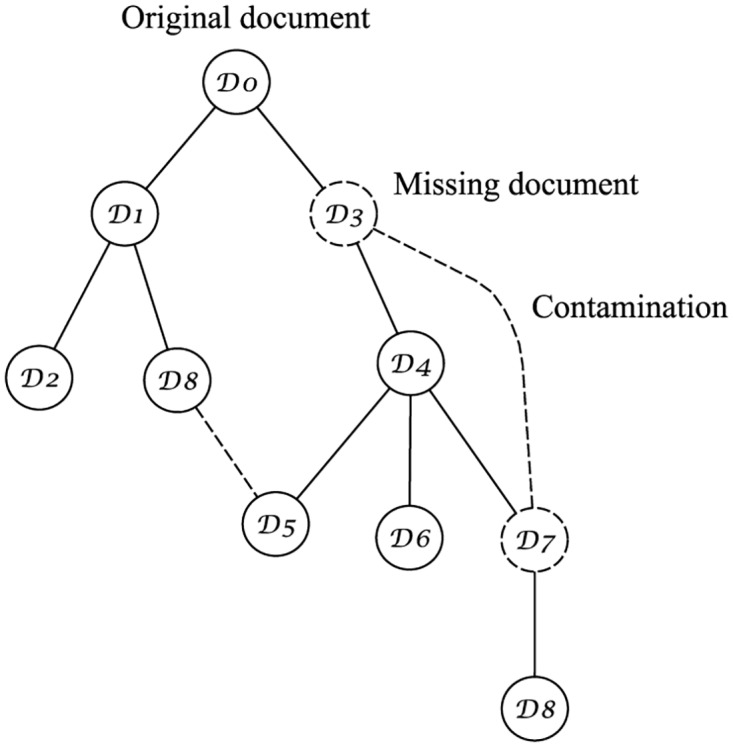
An example of a stemmatology tree. The original document $D_{0}$ may have several variants due to an erroneous copying or physical damage. In stemmatology, it is also important to consider the problem of missing documents (the dashed nodes), and contamination (dashed edges).

As previously mentioned, stemmatology also works with the idea of recovering text evolutionary trees, similar to what we have explored in text phylogeny. However, while both approaches try to solve the problem of genealogical relationships, stemmatology mainly targets manuscripts, whose variants, in most of the cases, were unintentionally created. For instance, some parts of an original document $D_{0}$ may be lost due to physical damage, or errors can be introduced by scribes while copying $D_{0}$. These errors can also propagate, in different times, and by different scribes, originating several variants of $D_{0}$ in a tree structure similar to the one Fig 10 depicts.

In stemmatology, aside from trying to find the relationship among the variants, there are two other problems to be solved during the tree reconstruction: *missing documents*, and *contamination*. In Fig 10, we represented the former case by the dashed nodes $D_{3}$ and $D_{7}$, and the latter case by the dashed edges ($D_{8},D_{5}$), and ($D_{3},D_{7}$). Contamination may happen due to different reasons: when the scribe copied the manuscript using several exemplars simultaneously, or used different exemplars for each part of his/her transcriptions, sometimes to verify or to improve it, by erasing former readings or by creating new ones [63].

Although the approach proposed in this paper do not target stemmatology applications directly, we performed a last experiment by applying it in two stemmatology datasets, which were previously used in a computer-assisted stemmatology challenge [15, 64]:

- *Parzival* dataset: This dataset comprises 16 documents with the beginning of the German poem *Parzival* by Wolfram von Eschenbach, translated to English by A.T. Hatto, and copied by hand.
- *Heinrich* dataset: It was artificially constructed by volunteer scribes, who copied a given text by hand, following an imaginary stemma. It consists of sixty-seven variants of a text written in old Finnish (*Piispa Henrikin surmavirsi—’The Death-Psalm of Bishop Henry’)*, which is approximately 1,200 words long. However, thirty of the text variants were intentionally left out of the dataset, to simulate the scenario where some of the manuscritps are missing. Some of the manuscripts also had some significant passages deleted to simulate the cases where they were partially destroyed.

**Fig 11:**
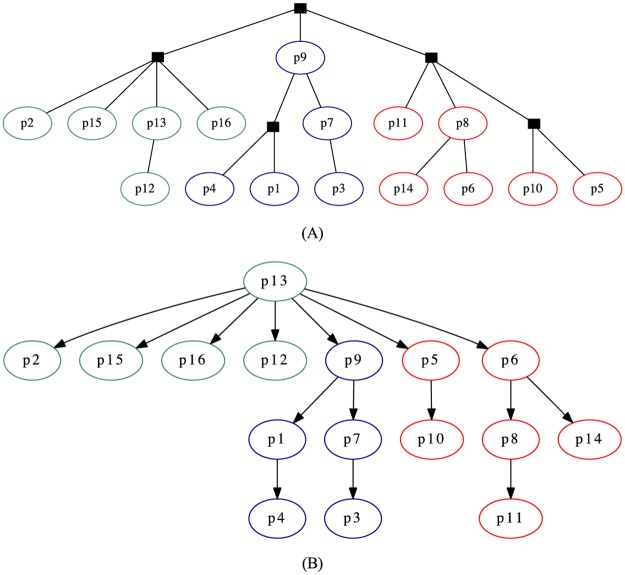
Reconstructed tree for *Parzival* dataset. (A) The ground-truth stemma [15, 64]. To help comparison with the reconstructed trees by the proposed approach, groups of nodes were separated by color. The black squares represent the missing documents. (B) Reconstructed tree using tf-idf (word-based 1-, 2-, 3-grams).

In both datasets, the dissimilarity between the documents were calculated using tf-idf (1-, 2-, 3-grams), and the reconstruction was performed using the minimum cost approach (Section Materials and Methods). Fig 11(A) shows the ground-truth stemma for the *Parzival* dataset [15, 64]. This ground-truth also includes the five documents that simulate the missing manuscripts. They are represented by the black squares in the tree. The documents considered the surviving variants are shown labeled with letters and numbers, and grouped by colors to help the comparison with the trees reconstructed by the proposed approach in Fig 11(B).

It is worth noting that the method proposed in this paper considers only the documents present in the set. Therefore, if two documents come from a single ancestor, but this ancestor is a missing document, the two documents are placed on the same level of the tree. In this case, the tree is reconstructed considering which arrangement of the given documents return a tree with the lowest cost, sometimes placing one of the documents as the parent of the other, for instance. This can be visualized with nodes p2/p15/p13/p16: since their common ancestor is not present in the set we analysed, node p13 is placed as the parent of the other nodes in the reconstructed tree in Fig 11(B). The same happens with the set of nodes p1/p4 and p5/p10. Thus, the tree configuration appears different as in the ground-truth, since it does not locate the missing nodes on the reconstructed tree. Nonetheless, it is able to correctly group the documents that are closer to each other, as shown in Fig 11(B).

**Fig 12:**
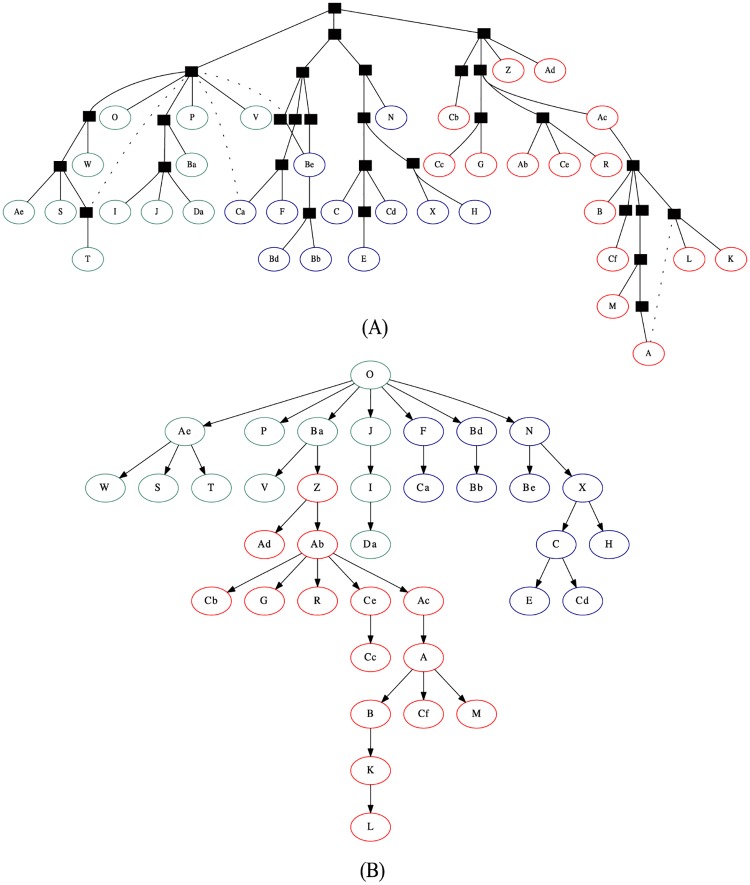
Reconstructed tree for *Heinrich* dataset. (A) The ground-truth stemma as provided by the computer-assisted stemmatology challenge [15, 64]. To help comparison with the reconstructed trees by the proposed approach, groups of nodes were separated by color. The black squares represent the thirty missing documents that were left out of the dataset. (B) Reconstructed tree using tf-idf (word-based 1-, 2-, 3-grams).

A similar behavior can be visualized in Fig 12, for *Heinrich* dataset [15, 64]. In this case, the ground-truth tree (Fig 12(A)) also includes the thirty documents that simulate the missing manuscripts, and the dashed edges indicates the contamination of a copy. Results are similar to the ones obtained in *Parzival* dataset, in which the nodes appear separated by color. Although a quantitative evaluation is diffcult in both datasets, due to the presence of missing nodes in the ground-truth, our approach can correctly group the nodes, which can be useful for a further analysis made by a stemmatology expert.

## Conclusions and Future Work

In this paper, we proposed a framework for reconstructing text evolutionary trees, aiming at reconstructing the history of modifications that a set of related documents has gone through. It is far from being a closed solution for the text phylogeny problem, as there are many variables to consider depending on the target set of documents. However, unlike previous approaches, the proposed framework does not need to take into account any side information (e.g., documents’ time of publication), neither assumptions regarding directionality in the documents’ contents (e.g., giving weights to some operations, or textual features) to solve the parent-child relationship. Nonetheless, when available, this knowledge can be easily added to the framework.

We compared the performance of three different methods for calculating the dissimilarity between the documents, with tf-idf presenting the best results in terms of computational efficiency and results effectiveness when dealing with balanced trees. We have also evaluated three different approaches for reconstructing the phylogeny tree, which can be used according to what is known about the target set of documents. In this sense, in our proposed framework, we divided the text phylogeny problem into three distinct scenarios: (i) when the tree orientation can be learned but not presumed, (ii) when the tree orientation can be presumed, but not learned, and finally, (iii) when the tree orientation can neither be presumed nor learned.

The first case happens when it is not possible to make assumptions over the directionality of the tree, but a training set is available such that the trees’ orientation can be inferred by a machine learning algorithm. We showed that only a few training examples are needed to reasonably learn which textual features point to the class of the documents. Although we used only content related features (the documents’ terms), features such as differences in the documents’ vocabulary richness, and the presence of automated reuse were useful to classify a document as being original or reused. For the vocabulary richness, this is particularly true for *Wikipedia* dataset, as the first revision is generally smaller and carries less content than the others. For the automated reuse, bots that perform text reuse are prone to use some terms more frequently than humans. In the synthetic dataset, although we use two comprehensive corpora (WordNet and COCA N-grams) to create a realistic language model, the automated reuse can give hints about the classes of the documents. We plan to expand our current approach and include other features that are not extracted from the text (such as number of edges of a given document and the average distance from a given document to its adjacent nodes) in a future work.

In the second case, there is no training set available, but we can make hypotheses or use knowledge from an expert in the area about the underlying branching process that generated the trees. When the branching process is random and produces, on average, balanced trees (such as the synthetic Reuters data or the real *Heinrich* dataset), we proposed a simple heuristic that can reasonably reconstruct the original TPT’s, as evidenced in Tables 3 and 4 in the *Experiments and Results section*.

Finally, in the last case, when there are no training examples available and we cannot hypothesize anything about the branching process (which is true in most of the cases for text phylogeny), we showed that it it possible to reliably build a non-oriented tree that gives an initial hint about the existing relationship among the documents. Evaluation with the Indirect Edges metric showed that the non-oriented tree reconstructed by the proposed framework (especially when the dissimilarities are computed through tf-idf) approximates well the real phylogeny tree. Although it is hard to infer the root of the tree without having any assumption either about the documents’ relationship or any training samples, the initial hypothesis provided by the non-oriented tree can narrow down the number of possible relationships among the documents. With this information, a forensic expert or a stemmatology researcher, for instance, can have a starting point for deciding which is most likely the correct phylogeny tree.

Furthermore, we showed the application of our framework in two potential application areas: plagiarism and stemmatology. Although for each of these problems it is necessary to make some modifications in the current framework, we were able to obtain satisfactory results without adapting the framework to these application domains. In plagiarism, one of the main concerns lies on distinguishing which documents are the result of real plagiarism, and which have only topic similarity. Regarding the application of our framework in stemmatology, although we are not able to identify the missing nodes in the stemma, we can nearly recover the tree structure, and the relationship among the nodes within the set being analyzed. Currently, our approach does not take into account the inclusion of unknown intermediate internal vertices and edges as in a Steiner tree, but we do acknowledge they may represent objects that can be helpful to better represent the phylogeny. This is an interesting variation of the phylogeny problem we have been working with and it will be considered in a future work, which could also be helpful to solve the problem of the connection between missing nodes in the stemmatology application. This can be a step forward to the development of new techniques in automatic analysis of the evolution of manuscripts. This can be a step forward to the development of new techniques in automatic analysis of the evolution of manuscripts.

As future work, other approaches to calculate the dissimilarity between the documents can be investigated, such as dynamic time warping, and state-of-the-art techniques used in plagiarism detection and related problems. We also intend to explore other types of transformations that can be used to generate the synthetic dataset. This way, we can test different text editing styles, and work with more challenging scenarios. We can also explore other types of tree topologies, with different levels of branching, for instance, in an attempt to create a more diversified dataset. In addition, we plan to explore other application areas that could benefit from our framework, such as source-code plagiarism, implementing the necessary modifications to make it more robust.

## Supporting Information

S1 File This file contains all supporting Figs(A-B) and Tables(A-I). Fig A in S1 File. Comparison of the average of progressive editing limit results for all metrics combined with the minimum-cost heuristic-based approach. The reconstruction using tf-idf (1-, 2-, 3-grams) had the best results, followed by the normalized edit distance. **Fig B in S1 File.** Progressive editing limit results for all metrics considering the best performing dissimilarity func- tion, tf-idf (1-, 2-, 3-grams). The plots show a small variation of accuracy with respect to the tree sizes, except for the Directed Edges case, with the results improving as the number of nodes increases. **Table A in S1 File.** Number of linear trees in Wikipedia dataset. **Table B in S1 File.** Percentage of child nodes *c* per tree size |*T*| in the Wikipedia dataset. **Table C in S1 File.** Average of the edit distance between parent and child nodes in Wikipedia dataset. **Table D in S1 File.** Percentage of edges that were edited in each editing interval in Wikipedia dataset. **Table E in S1 File.** Neighbor probability for all dissimilarity functions combined with the minimum cost approach. Best results are highlighted in bold. **Table F in S1 File.** Results for the minimum cost heuristic-based reconstruction considering the progressive editing limit case in the synthetic dataset, and editing limit L = 50%. Best results were achieved by tf-idf, highlighted in blue. **Table G in S1 File.** Results for the minimum cost heuristic-based reconstruction considering the mixed editing limit case in the synthetic dataset, and L randomly chosen in the interval I = 5%, 10%, …, 50%. In this case, tf-idf also outperformed the other approaches in all tree sizes and metrics. **Table H in S1 File.** Results for the minimum cost heuristic-based reconstruction for Wikipedia dataset. In this case, the edit distance variants presented the best results, as tf-idf cannot capture some subtle modifications in Wikipedia, such as in the ordering of words. In particular, tf-idf presented the best result for Depth metric. **Table I in S1 File.** Tree reconstruction using Random Forests. After several tests, we obtained the best results with 500 estimators and information gain as split quality criterion.
(PDF)
