# Supplementary material for: On the Reconstruction of Text Phylogeny Trees: Evaluation and Analysis of Textual Relationships
Source: PLoS One. 2016 Dec 19;11(12):e0167822. doi: 10.1371/journal.pone.0167822 (PMC5167249; doi:10.1371/journal.pone.0167822)
Supplement: S1 File — Fig A in S1 File. Comparison of the average of progressive editing limit results for all metrics combined with the minimum-cost heuristic-based approach. The reconstruction using tf-idf (1-, 2-, 3-grams) had the best results, followed by the normalized edit distance. Fig B in S1 File. Progressive editing limit results for all metrics considering the best performing dissimilarity func- tion, tf-idf (1-, 2-, 3-grams). The plots show a small variation of accuracy with respect to the tree sizes, except for the Directed Edges case, with the results improving as the number of nodes increases. Table A in S1 File. Number of linear trees in Wikipedia dataset. Table B in S1 File. Percentage of child nodes c per tree size |T| in the Wikipedia dataset. Table C in S1 File. Average of the edit distance between parent and child nodes in Wikipedia dataset. Table D in S1 File. Percentage of edges that were edited in each editing interval in Wikipedia dataset. Table E in S1 File. Neighbor probability for all dissimilarity functions combined with the minimum cost approach. Best results are highlighted in bold. Table F in S1 File. Results for the minimum cost heuristic-based reconstruction considering the progressive editing limit case in the synthetic dataset, and editing limit L = 50%. Best results were achieved by tf-idf, highlighted in blue. Table G in S1 File. Results for the minimum cost heuristic-based reconstruction considering the mixed editing limit case in the synthetic dataset, and L randomly chosen in the interval I = 5%, 10%, …, 50%. In this case, tf-idf also outperformed the other approaches in all tree sizes and metrics. Table H in S1 File. Results for the minimum cost heuristic-based reconstruction for Wikipedia dataset. In this case, the edit distance variants presented the best results, as tf-idf cannot capture some subtle modifications in Wikipedia, such as in the ordering of words. In particular, tf-idf presented the best result for Depth metric. Table I in S1 File [file pone.0167822.s001.pdf]

Supplementary material for

On The Reconstruction of Text Phylogeny Trees: Analysis and Evaluation of Textual Relationships

PlosOne submission

Guilherme D. Marmerola, Marina A. Oikawa, Zanoni Dias,
Siome Goldenstein, and Anderson Rocha

1 Dataset construction

In this section, we present more details regarding the parameters and procedure used to construct the Synthetic and *Wikipedia* datasets. For both datasets, the text files, the ground-truth, the phylogeny reconstructed by our algorithm, and the tree structures are available at `http://www.recod.ic.unicamp.br/~oikawa/datasets.html#text`.

1.1 Synthetic dataset

In our algorithm for generating the trees in the synthetic dataset, we used weights such that the operations are chosen with probability relative to these weights. Through empiric experiments, the weights set to the operations were as listed below:

- Synonym substitution: 2
- Typo insertion: 0.1
- Typo removal: 1
- Modifier insertion: 0.5
- Modifier removal: 0.1
- Sentence insertion: 0.1
- Sentence removal: 0.1

When one of the word operations above is chosen, only one word is modified at a time, counting as one editing operation. When the operation is performed in a sentence, we operate on multiple words, so the total count of editing operations is equal to the length (in words) of the sentence. In addition, to simulate sentence insertion, it is necessary to cache some sentences from the original text document, so when this operation is chosen, we have some sentences available to keep the text coherence. For this case, the proportion of held out sentences chosen was 0.2. The operations are performed until the editing limit L is reached, which varies in the interval $I = \{5\%, 10\%, ..., 50\%\}$, in steps of 5%. Algorithm 1 (*Experiments* section) summarizes the construction of our synthetic dataset.

1.2 *Wikipedia* dataset

Regarding the articles in *Wikipedia* dataset, we collected them from the **Featured Articles** category available up to March 2014, disregarding changes to references, table of contents, and images. Furthermore, as mentioned in Section 4.1.2, in our *Wikipedia* dataset, we considered not only linear trees, but also trees with branchings by applying the *revision revert algorithm* (Algorithm 2 in the manuscript). To give a general idea about the structure of the trees of this dataset, we have performed an analysis of the number of linear and non-linear trees included in the dataset, as shown on Table A:

It is important to notice that in this dataset, the number of linear trees depends on the number of nodes in it, since the higher the number of trees, higher are the chances that there is a reversion, and therefore, a branching in the tree. Thus, as shown in Table A, for trees with 10 nodes, there is a large number of linear trees, and this number decreases as the number of nodes increases. In our dataset, within a set of 304 trees with 400 nodes each, there is only one linear tree. Furthermore, in Table B, we show the number of children nodes per node in the entire dataset to show the distribution of the nodes in the branchings. Although most of nodes have only one child node, we can see some variation. For instance, there is a case of one node with 38 children nodes for a tree with 400 documents. We included this additional information about the construction of the dataset in the manuscript's supplementary material along with the dataset's description.

Table A: Number of linear trees in Wikipedia dataset

$\|T\|$	Number of linear trees	%	Total number of trees
10	633	73.70%	859
20	469	54.60%	859
30	345	40.16%	859
40	270	31.47%	858
50	218	25.53%	854
100	87	11.40%	763
200	23	4.02%	572
300	6	1.43%	419
400	1	0.33%	304

Table B: Percentage of child nodes c per tree size $|T|$ in the Wikipedia dataset

c \ $\|T\|$	10	20	30	40	50	100	200	300	400
**1**	96.489%	96.148%	95.720%	95.482%	95.382%	94.843%	93.535%	92.213%	91.562%
**2**	3.081%	3.132%	3.386%	3.538%	3.556%	3.756%	4.372%	5.049%	5.456%
**3**	0.377%	0.534%	0.613%	0.622%	0.645%	0.800%	1.117%	1.420%	1.506%
**4**	0.040%	0.122%	0.145%	0.188%	0.218%	0.263%	0.404%	0.539%	0.600%
**5**	0.013%	0.026%	0.060%	0.080%	0.090%	0.137%	0.201%	0.272%	0.307%
**6**	-	0.026%	0.021%	0.016%	0.036%	0.067%	0.129%	0.167%	0.178%
**7**	-	0.013%	0.038%	0.035%	0.033%	0.057%	0.069%	0.114%	0.114%
**8**	-	-	0.013%	0.026%	0.023%	0.029%	0.054%	0.069%	0.076%
**9**	-	-	-	0.006%	0.010%	0.016%	0.033%	0.038%	0.055%
**10**	-	-	0.004%	0.006%	0.008%	0.011%	0.022%	0.035%	0.038%
**11**	-	-	-	-	-	0.004%	0.012%	0.020%	0.030%
**12**	-	-	-	-	-	0.003%	0.011%	0.013%	0.020%
**13**	-	-	-	-	-	0.003%	0.010%	0.011%	0.013%
**14**	-	-	-	-	-	0.004%	0.006%	0.008%	0.013%
**15**	-	-	-	-	-	0.000%	0.003%	0.005%	0.005%
**16**	-	-	-	-	-	0.001%	0.004%	0.004%	0.006%
**17**	-	-	-	-	-	-	0.007%	0.006%	0.006%
**18**	-	-	-	-	-	-	0.002%	0.003%	0.003%
**19**	-	-	-	-	-	-	0.001%	0.001%	0.001%
**20**	-	-	-	-	-	-	0.003%	0.005%	0.005%
**21**	-	-	-	-	-	0.001%	0.002%	0.002%	0.002%
**22**	-	-	-	-	-	-	0.002%	0.002%	0.002%
**24**	-	-	-	-	-	-	0.000%	0.002%	0.001%
**25**	-	-	-	-	-	0.001%	0.002%	0.002%	0.001%
**27**	-	-	-	-	-	0.001%	-	-	-
**30**	-	-	-	-	-	-	0.001%	-	-
**38**	-	-	-	-	-	-	-	-	0.001%

We have also performed two additional experiments to provide more details about the *Wikipedia* dataset characteristics. In Table C, we present the results for the average edit distance between parent and child nodes in number of words for $|T| \in \{10, 20, 30, 40, 50\}$. With these results, we intended to show that the similarity of the documents present in the dataset are very high, making the inference of the phylogeny trees more difficult. The editing percentage in the last column shows the average of the distance between parent and child nodes, normalized by the number of words in the parent's document (to compensate effects on the score when the documents do not have similar length).

To complement the results in Table C, we show in Table D, for each tree size, the number of edges that were edited according to the editing interval in the first column. For instance, for trees with 30 nodes ($|T|$=30), we can see that 93.126% of the edges have an editing amount between 0.0% and 0.5%, while only 0.197% edges have 95.0% to 100.0% of editing amount.

Table C: Average of the edit distance between parent and child nodes in *Wikipedia* dataset

$\|T\|$	**Avg. of Text Size (# of words)**	**Avg. Edit Distance**	**Editing Percentage**
10	660.69	73.99	2.44%
20	858.26	67.86	1.36%
30	1024.80	66.92	0.96%
40	1176.54	66.60	0.73%
50	1314.58	65.16	0.61%

Table D: Percentage of edges that were edited in each editing interval in *Wikipedia* dataset

**Editing Interval**	$\|T\| = 10$	$\|T\| = 20$	$\|T\| = 30$	$\|T\| = 40$	$\|T\| = 50$
**0.0% - 0.5%**	86.509%	91.194%	93.126%	94.387%	95.15%
**0.5 % - 1.0%**	5.937%	4.317%	3.629%	3.048%	2.69%
**1.0% - 1.5%**	1.876%	1.128%	0.854%	0.676%	0.58%
**1.5% - 2.0%**	1.048%	0.650%	0.499%	0.387%	0.33%
**2.0% - 2.5%**	0.660%	0.429%	0.290%	0.237%	0.20%
**2.5% - 3.0%**	0.427%	0.276%	0.205%	0.168%	0.14%
**3.5% - 4.0%**	0.323%	0.184%	0.137%	0.105%	0.09%
**4.0% - 4.5%**	0.336%	0.202%	0.137%	0.105%	0.09%
**4.5% - 5.0%**	0.155%	0.080%	0.060%	0.048%	0.04%
**5.0% - 5.5%**	0.246%	0.135%	0.097%	0.081%	0.07%
**5.5% - 6.0%**	0.155%	0.086%	0.060%	0.051%	0.04%
**6.0% - 6.5%**	0.129%	0.080%	0.052%	0.039%	0.03%
**6.5% - 7.0%**	0.103%	0.067%	0.044%	0.036%	0.03%
**7.0% - 7.5%**	0.103%	0.061%	0.040%	0.030%	0.02%
**7.5% - 8.0%**	0.052%	0.031%	0.020%	0.018%	0.01%
**8.0% - 8.5%**	0.116%	0.067%	0.044%	0.036%	0.03%
**8.5% - 9.0%**	0.039%	0.018%	0.012%	0.012%	0.01%
**9.0% - 9.5%**	0.013%	0.006%	0.004%	0.003%	0.00%
**9.5% - 10.0%**	0.065%	0.037%	0.032%	0.027%	0.02%
**10.0% - 15.0%**	0.013%	0.006%	0.004%	0.003%	0.00%
**15.0% - 20.0%**	0.453%	0.245%	0.173%	0.135%	0.11%
**20.0% - 25.0%**	0.194%	0.110%	0.077%	0.060%	0.05%
**25.0% - 30.0%**	0.116%	0.067%	0.044%	0.036%	0.03%
**30.0% - 35.0%**	0.103%	0.049%	0.036%	0.027%	0.03%
**35.0% - 40.0%**	0.052%	0.031%	0.016%	0.015%	0.01%
**40.0% - 45.0%**	0.026%	0.025%	0.016%	0.012%	0.01%
**45.0% - 50.0%**	0.052%	0.025%	0.016%	0.009%	0.01%
**50.0% - 55.0%**	0.039%	0.018%	0.012%	0.009%	0.01%
**55.0% - 60.0%**	0.013%	0.006%	0.004%	0.003%	0.00%
**60.0% - 65.0%**	0.039%	0.018%	0.012%	0.012%	0.01%
**65.0% - 70.0%**	0.026%	0.012%	0.008%	0.006%	0.01%
**70.0% - 75.0%**	0.013%	0.006%	0.004%	0.003%	0.00%
**75.0% - 80.0%**	0.039%	0.018%	0.012%	0.009%	0.01%
**80.0% - 85.0%**	0.013%	0.006%	0.004%	0.003%	0.00%
**85.0% - 90.0%**	0.013%	0.012%	0.008%	0.006%	0.01%
**90.0% - 95.0%**	0.026%	0.018%	0.012%	0.009%	0.01%
**95.0% - 100.0%**	0.479%	0.276%	0.197%	0.147%	0.12%

2 Experiments Results

In this section, we present additional experiments performed in the Synthetic and *Wikipedia* dataset.

2.1 Minimum-cost heuristic

Table E shows the number of times (%) that the reconstructed root or one of its neighbors was the actual root (Neighbor probability), for all dissimilarity functions combined with the minimum-cost heuristic, and considering $L = 50\%$. These results complement the results shown in Table 1 of the submitted manuscript.

Table E: Neighbor probability for all dissimilarity functions combined with the minimum cost approach. Best results are highlighted in bold.

	Unnormalized edit distance		Normalized edit distance	
$\lvert T\rvert$	**Neighbor probability**	**Neighbor set size**	**Neighbor probability**	**Neighbor set size**
10	76.00%	4.41	76.50%	4.61
20	58.50%	4.82	68.50%	5.19
30	36.00%	4.79	70.50%	5.67
40	22.50%	4.63	64.00%	5.86
50	17.50%	4.83	64.00%	6.08

	NCD		tf-idf (1-,2-,3-grams)	
$\lvert T\rvert$	**Neighbor probability**	**Neighbor set size**	**Neighbor probability**	**Neighbor set size**
10	74.00%	4.42	**81.50%**	**4.67**
20	60.50%	4.90	**77.00%**	**5.34**
30	58.50%	5.34	**76.00%**	**5.91**
40	57.50%	5.36	**74.00%**	**6.14**
50	63.50%	5.47	**78.00%**	**6.55**

In Figure A, we show the average results (over tree sizes), for the progressive editing limit case of the synthetic dataset, for all dissimilarity functions, combined with the minimum-cost heuristic. Among all approaches, the reconstruction using tf-idf word-based 1-, 2-, 3-grams presented the best results, followed by the normalized edit distance. The unnormalized edit distance presented slightly better results than NCD up to $L = 35\%$, when the document length begins to become a relevant issue, as the transformations become more intense. These results also show the importance of normalization: while the remaining normalized methods managed to maintain robustness after reaching $L = 35\%$, the unnormalized edit distance results started to rapidly deteriorate. Nonetheless, the reconstruction of the undirected tree was nearly perfect for most of the L-range for all dissimilarity functions, as evidenced by the Indirect Edges metric (Figure A(a)). Figure A complements the graphics shown in Figure 6 of the submitted manuscript.

In Figure B, we show a detailed comparison over tree sizes, considering the best performing dissimilarity function, tf-idf. The plots reveal a small variation of accuracy with respect to the tree sizes, with Directed Edges (Figure B(b)) being the metric with the most disperse results, improving as the number of trees increase. This is not unexpected: as the Roots metric is roughly constant over tree sizes, and Depth is close to 1, we expect that proportionately less wrong edges are present in larger trees.

To finish the analysis of the synthetic case with progressive editing limit, we present the results considering editing limit $L = 50\%$ in Table F, divided by tree size. In this case, tf-idf also outperformed the other methods in all tree sizes and metrics.

For the synthetic case with mixed editing limit, the results are also promising, showing robustness of the proposed approach when reconstructing trees composed of a non-homogeneous combination of mildly ($L \leq 25\%$) or heavily ($25\% \leq L \leq 50\%$) modified documents. In agreement with the progressive editing limit case, the best results were achieved by tf-idf word-based 1-, 2-, 3-grams as presented in Table G. Table F and Table G complement the results in Table 2 of the submitted manuscript.

Lastly, in Table H, we present the results for all metrics combined with the minimum cost approach for the *Wikipedia* dataset. It complements the results in Table 3 of the submitted manuscript.

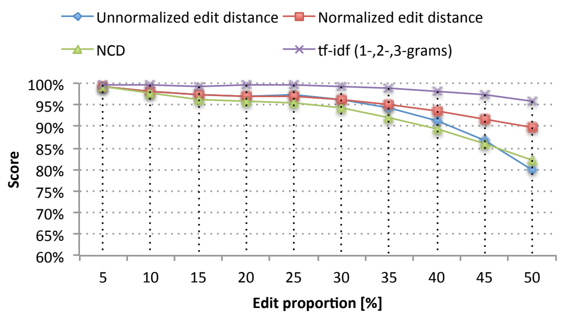

(a) Indirect edges

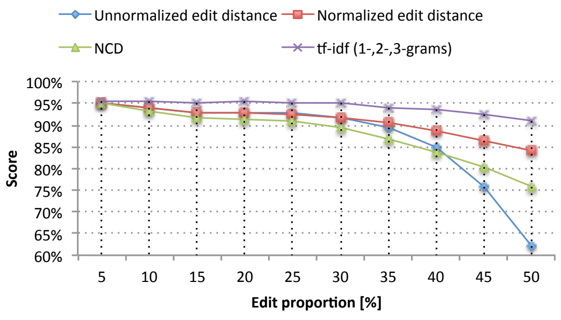

(b) Directed Edges

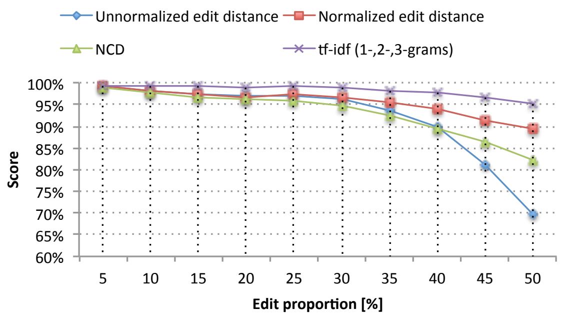

(c) Leaves

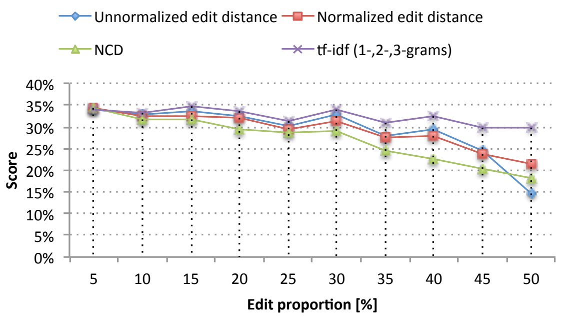

(d) Roots

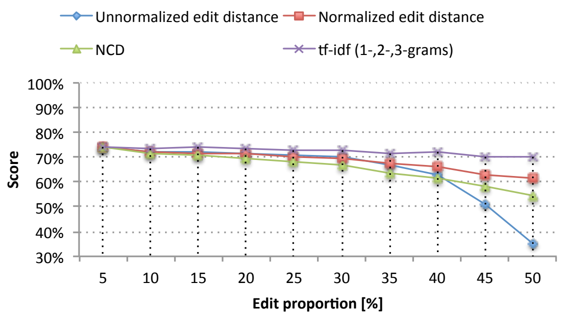

(e) Ancestry

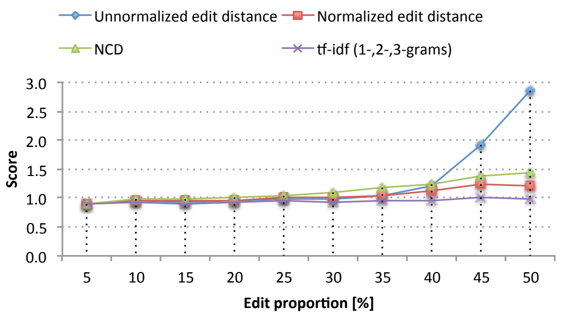

(f) Depth

Figure A: Comparison of the average of progressive editing limit results for all metrics combined with the minimum-cost heuristic-based approach. The reconstruction using tf-idf (1-, 2-, 3-grams) had the best results, followed by the normalized edit distance.

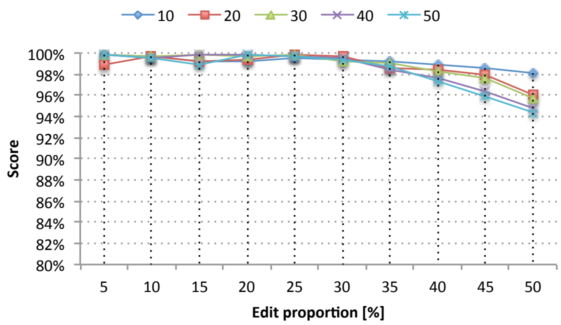

(a) Indirect edges

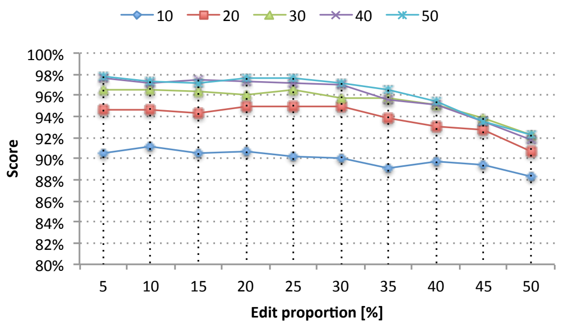

(b) Directed Edges

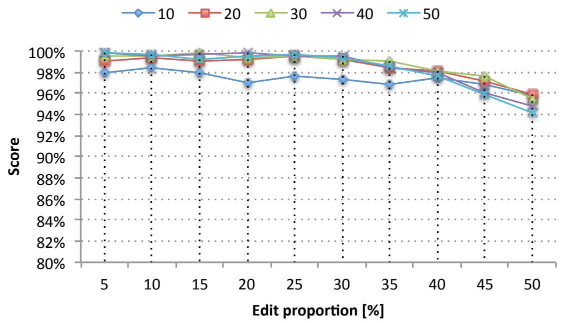

(c) Leaves

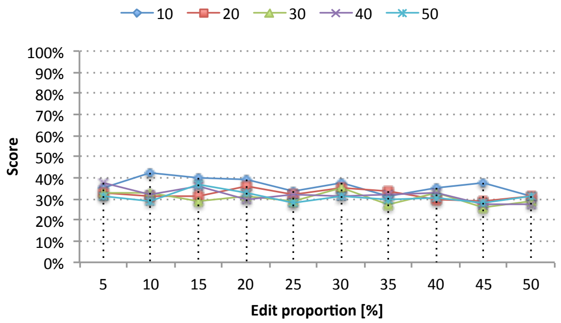

(d) Roots

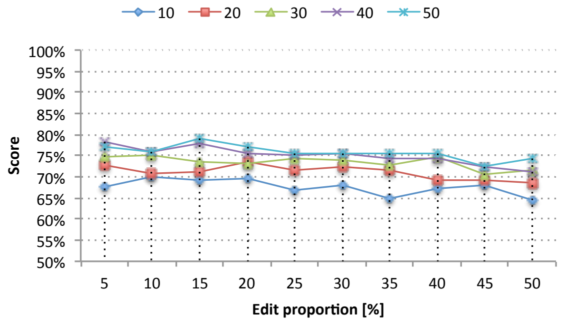

(e) Ancestry

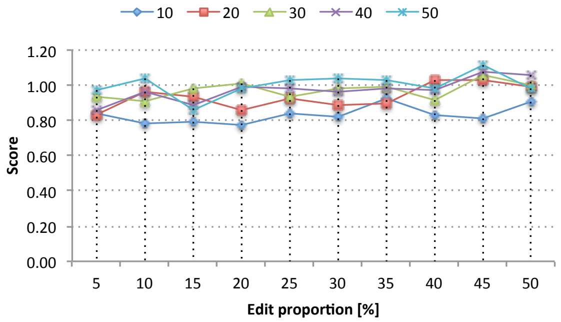

(f) Depth

Figure B: Progressive editing limit results for all metrics considering the best performing dissimilarity function, tf-idf (1-, 2-, 3-grams). The plots show a small variation of accuracy with respect to the tree sizes, except for the Directed Edges case, with the results improving as the number of nodes increases.

Table F: Results for the minimum cost heuristic-based reconstruction considering the **progressive** editing limit case in the synthetic dataset, and editing limit $L = 50\%$. Best results were achieved by tf-idf, highlighted in blue.

	Unnormalized edit distance						Normalized edit distance					
$\|T\|$	**Ind.Edges**	**Dir.Edges**	**Leaves**	**Roots**	**Ancestry**	**Depth**	**Ind.Edges**	**Dir.Edges**	**Leaves**	**Roots**	**Ancestry**	**Depth**
10	90.40%	78.40%	86.90%	24.50%	56.00%	1.07	92.80%	82.00%	90.90%	22.00%	57.30%	1.04
20	82.90%	70.10%	76.10%	24.50%	48.60%	1.67	90.80%	84.90%	90.90%	25.50%	62.10%	1.16
30	77.80%	59.00%	65.30%	14.00%	31.20%	2.83	89.60%	85.30%	89.70%	21.00%	62.80%	1.26
40	74.60%	52.60%	61.30%	4.00%	21.70%	4.04	88.00%	84.50%	88.40%	18.50%	62.00%	1.36
50	73.40%	49.70%	59.00%	6.00%	18.30%	4.67	87.00%	83.80%	86.40%	20.00%	62.60%	1.29

	NCD						tf-idf (1-,2-,3-grams)					
$\|T\|$	**Ind.Edges**	**Dir.Edges**	**Leaves**	**Roots**	**Ancestry**	**Depth**	**Ind.Edges**	**Dir.Edges**	**Leaves**	**Roots**	**Ancestry**	**Depth**
10	89.10%	77.90%	86.00%	25.00%	55.50%	1.08	**98.10%**	**88.30%**	**95.80%**	**31.00%**	**64.50%**	**0.91**
20	82.40%	75.70%	83.50%	19.00%	54.50%	1.36	**96.00%**	**90.70%**	**95.90%**	**31.50%**	**68.60%**	**1.00**
30	81.30%	76.20%	81.90%	15.00%	54.70%	1.54	**95.70%**	**92.20%**	**95.50%**	**29.00%**	**71.40%**	**1.00**
40	79.10%	74.60%	80.10%	15.50%	53.50%	1.62	**94.80%**	**91.80%**	**94.80%**	**27.50%**	**71.10%**	**1.06**
50	78.70%	74.90%	78.80%	16.50%	54.70%	1.54	**94.40%**	**92.20%**	**94.10%**	**31.00%**	**74.20%**	**0.98**

Table G: Results for the minimum cost heuristic-based reconstruction considering the **mixed** editing limit case in the synthetic dataset, and L randomly chosen in the interval $I = \{5\%, 10\%, ..., 50\%\}$. In this case, tf-idf also outperformed the other approaches in all tree sizes and metrics.

	Unnormalized edit distance						Normalized edit distance					
$\|T\|$	**Ind.Edges**	**Dir.Edges**	**Leaves**	**Roots**	**Anc.**	**Depth**	**Ind.Edges**	**Dir.Edges**	**Leaves**	**Roots**	**Anc.**	**Depth**
10	92.50%	81.60%	90.20%	28.90%	59.30%	1.00	91.80%	80.50%	89.70%	28.20%	57.70%	1.05
20	89.80%	83.70%	89.40%	25.80%	61.30%	1.12	89.30%	83.20%	89.80%	24.70%	59.90%	1.18
30	88.40%	83.50%	88.10%	24.10%	60.30%	1.30	88.60%	84.00%	89.70%	22.90%	60.20%	1.32
40	87.70%	83.70%	87.60%	24.40%	61.20%	1.32	88.00%	84.40%	89.30%	22.70%	61.10%	1.35
50	87.20%	83.60%	87.00%	24.90%	60.90%	1.37	88.00%	85.00%	89.70%	23.10%	61.50%	1.40

	NCD						tf-idf (1-,2-,3-grams)					
$\|T\|$	**Ind.Edges**	**Dir.Edges**	**Leaves**	**Roots**	**Anc.**	**Depth**	**Ind.Edges**	**Dir.Edges**	**Leaves**	**Roots**	**Anc.**	**Depth**
10	89.70%	77.90%	87.00%	24.30%	54.50%	1.11	**97.00%**	**86.90%**	**95.30%**	**32.20%**	**63.90%**	**0.92**
20	87.80%	81.10%	87.90%	20.60%	57.00%	1.26	**96.90%**	**91.70%**	**97.00%**	**29.20%**	**68.50%**	**0.99**
30	86.90%	81.80%	87.50%	19.90%	57.40%	1.41	**96.60%**	**93.00%**	**97.20%**	**28.70%**	**70.30%**	**1.05**
40	86.40%	82.10%	86.50%	20.20%	57.90%	1.47	**96.60%**	**93.70%**	**96.90%**	**27.00%**	**71.30%**	**1.09**
50	86.30%	82.70%	86.80%	22.00%	59.40%	1.46	**96.50%**	**94.20%**	**97.10%**	**30.40%**	**73.40%**	**1.07**

Table H: Results for the minimum cost heuristic-based reconstruction for *Wikipedia* dataset. In this case, the edit distance variants presented the best results, as tf-idf cannot capture some subtle modifications in *Wikipedia*, such as in the ordering of words. In particular, tf-idf presented the best result for Depth metric.

	Unnormalized edit distance						Normalized edit distance					
$\|T\|$	**Ind.Edges**	**Dir.Edges**	**Leaves**	**Roots**	**Anc.**	**Depth**	**Ind.Edges**	**Dir.Edges**	**Leaves**	**Roots**	**Anc.**	**Depth**
10	**87.90%**	**44.90%**	**39.80%**	0.58%	**21.60%**	3.57	87.80%	44.10%	39.10%	**0.70%**	21.00%	3.60
20	87.50%	**45.40%**	**35.30%**	0.12%	**19.80%**	7.10	**87.60%**	44.60%	35.00%	**0.23%**	19.20%	7.17
30	87.50%	**46.30%**	**34.10%**	**0.70%**	**19.30%**	10.50	**87.70%**	45.40%	33.80%	0.23%	18.70%	10.60
40	87.80%	**47.30%**	**33.70%**	**0.35%**	**19.30%**	13.80	**87.90%**	46.40%	33.90%	**0.35%**	18.80%	14.00
50	88.00%	**47.60%**	**33.70%**	**0.23%**	**19.20%**	17.20	**88.20%**	46.80%	33.70%	0.12%	18.60%	17.40

	NCD						tf-idf (1-,2-,3-grams)					
$\|T\|$	**Ind.Edges**	**Dir.Edges**	**Leaves**	**Roots**	**Anc.**	**Depth**	**Ind.Edges**	**Dir.Edges**	**Leaves**	**Roots**	**Anc.**	**Depth**
10	80.30%	40.30%	32.10%	0.47%	19.70%	3.40	80.50%	39.20%	32.20%	0.12%	18.60%	**3.26**
20	79.00%	39.70%	26.50%	0.00%	17.50%	6.76	78.80%	38.80%	25.40%	0.00%	16.80%	**6.02**
30	78.40%	39.70%	23.40%	0.23%	16.90%	9.91	78.20%	38.60%	23.20%	0.00%	15.70%	**8.67**
40	78.40%	40.40%	22.60%	0.00%	16.90%	13.10	78.40%	39.10%	22.70%	0.00%	15.60%	**11.30**
50	78.40%	40.50%	22.20%	0.12%	16.80%	16.20	78.20%	39.10%	22.40%	0.00%	15.40%	**13.80**

2.2 Supervised Machine Learning

Table I shows the results using Random Forests for the synthetic and the *Wikipedia* datasets. In comparison to SVMs, we had similar performance for the synthetic data, and obtained improvements for *Wikipedia trees*. The accuracy for the Roots metric was above 45% for all tree sizes, along with moderate improvements in Directed Edges, Ancestry and Depth metrics. These results complement the results presented in Table 5 of the submitted manuscript.

Table I: Tree reconstruction using Random Forests. After several tests, we obtained the best results with 500 estimators and information gain as split quality criterion.

	Synthetic dataset						*Wikipedia* dataset					
$\|T\|$	**Ind.Edges**	**Dir.Edges**	**Leaves**	**Roots**	**Anc.**	**Depth**	**Ind.Edges**	**Dir.Edges**	**Leaves**	**Roots**	**Anc.**	**Depth**
10	98.90%	98.60%	99.10%	97.40%	98.00%	0.03	88.40%	64.60%	54.40%	54.70%	61.10%	1.87
20	98.50%	98.20%	98.50%	98.20%	97.20%	0.02	85.00%	63.40%	36.10%	47.10%	58.10%	2.42
30	97.90%	97.70%	98.60%	97.80%	97.60%	0.02	88.40%	69.50%	35.70%	51.70%	65.40%	2.46
40	98.00%	97.80%	98.30%	97.20%	97.00%	0.03	87.70%	71.40%	36.00%	53.60%	66.20%	2.37
50	98.00%	97.80%	98.20%	97.40%	97.10%	0.03	88.10%	70.10%	35.10%	45.90%	65.00%	3.79
